# The Efficacy and Safety of Probiotics for Allergic Rhinitis: A Systematic Review and Meta-Analysis

**DOI:** 10.3389/fimmu.2022.848279

**Published:** 2022-05-19

**Authors:** Chao Luo, Shunlin Peng, Mao Li, Xudong Ao, Zhiqing Liu

**Affiliations:** Ear-Nose-Throat (E.N.T.) Department, Hospital of Chengdu University of Traditional Chinese Medicine, Chengdu, China

**Keywords:** probiotics, allergy rhinitis, allergy, systematic review, meta-analysis

## Abstract

**Background:**

Probiotics have proven beneficial in a number of immune-mediated and allergic diseases. Several human studies have evaluated the efficacy and safety of probiotics in allergic rhinitis; however, evidence for their use has yet to be firmly established.

**Objective:**

We undertook a systematic review and meta-analysis aiming to address the effect and safety of probiotics on allergic rhinitis.

**Methods:**

We systematically searched databases [MEDLINE (PubMed), Embase, and the Cochrane Central Register of Controlled Trials] from inception until June 1, 2021. Qualified literature was selected according to inclusion and exclusion criteria, the data were extracted, and a systematic review and meta-analysis was conducted.

**Results:**

Twenty-eight studies were included. The results showed that probiotics significantly relieved allergic rhinitis symptoms (standardized mean difference [SMD], −0.29, 95% confidence interval (CI) [−0.44, −0.13]; *p* = 0.0003, *I* ^2^ = 89%), decreased Rhinoconjunctivitis Quality of Life Questionnaire (RQLQ) scores compared with the control group (SMD, −0.64, 95% CI [−0.79, −0.49], *p* < 0.00001, *I* ^2^ = 97%), and increased T helper cell 1(Th1)/Th2 ratio (mean difference [MD], −2.47, 95% CI [−3.27, −1.68], *p* < 0.00001, *I* ^2^ = 72%). There was no significant change in overall or specific IgE levels between probiotic-treated and placebo-treated subjects (SMD, 0.09, 95% CI [−0.16, 0.34], *I* ^2^ = 0%, and SMD, −0.03, 95% CI [−0.18, 0.13], *p* = 0.72, *I* ^2^ = 0%, respectively).

**Conclusions:**

To sum up, probiotic supplement seems to be effective in ameliorating allergic rhinitis symptoms and improving the quality of life, but there is high heterogeneity in some results after subgroup analysis and clinicians should be cautious when recommending probiotics in treating allergic rhinitis.

**Systematic Review Registration:**

https://www.crd.york.ac.uk/PROSPERO/, PROSPERO (CRD42021242645).

## Introduction

Allergic rhinitis (AR) is characterized by a nasal sensitive inflammation, which is estimated to already affect 10%–40% of the worldwide population (1, 2). Common symptoms of AR are nasal itching, sneezing, rhinorrhea, and nasal congestion. In addition, some patients experience symptoms of allergic rhinoconjunctivitis, such as watery or itchy or red eyes. Severe AR can affect the quality of life, sleep, and work performance (1).

In 1989, Strachan found that the number of siblings was inversely related to the prevalence of hay fever among peers in the UK. Then, he proposed the “Hygiene hypothesis” (3), that the changed intestinal microbiota due to the lack of contact with infectious sources, parasites, and symbiotic microorganisms affects the normal development of immune system. The “Hygiene hypothesis” extends to the “Old Friends” and the “Microflora hypothesis” (4, 5). The “Microflora hypothesis” believes that a diverse gut microbiota plays an important role in shaping host immune development and that disruption or dysbiosis of the normal gut microbiota contributes to the development of immune disorders such as allergic diseases (6, 7). Host–microbes symbiosis plays a cardinal role in maintaining health and immune homeostasis. Changes in the intestinal flora are considered to be one of the most important indicators of allergic diseases (8, 9). Probiotics are live bacteria that colonize the gastrointestinal tract and they provide a health benefit to the host when administered in adequate amounts (10). Recent studies have shown that probiotics are non-pharmaceutical agents that can increase the production of systemic IFN, IL10, and IL12, improve the pre-Th1 immune response, and reduce Th2 cytokines (11), and thus have been proposed as modulators of the allergic response and advocated as therapeutic and preventive interventions for allergic disease (12, 13).

Probiotics include the Lactobacillus group (L. rhamnosus GG, L. sporogenes, L. reuteri RC-14, L. plantarum 299v, L. acidophilus, and L. lactis), the Bifidobacterium group (B. bifidum, B. longum, and B. infantis), the Streptococcus group (S. thermophilus, S. lactis, and S. fecalis), and non-bacterial organisms (non-pathogenic yeast Saccharomyces boulardii). The most common probiotics are the Lactobacillus and Bifidobacterium groups (14). Many studies have attempted to assess the role of probiotics in the treatment of AR with inconsistent findings. While some have found a protective effect of probiotics on AR (15–18), several others have found no association (19, 20). Given that there have been further published studies, we undertook a systematic review and meta-analysis aiming to address the effect and safety of probiotics on AR, and meanwhile, we attempted to explore the possible causes of between-study heterogeneity via subgroup.

## Methods and Analysis

### Study Registration

The protocol of this systematic review and meta-analysis has been registered on the PROSPERO platform with an assigned registration number CRD42021242645, based on the Preferred Reporting Items for Systematic Reviews and Meta-Analyses Protocols statement guidelines. This research was conducted based on this protocol.

### Database Search

We have performed a search in MEDLINE (PubMed), Embase, and the Cochrane Central Register of Controlled Trials. Additional studies will be sought by manually checking the references of included studies and relevant reviews. Searches will be restricted to publications appearing from inception to June 1, 2021. We used subject (“Rhinitis, Allergic”, “Rhinitis, Allergic, Seasonal”, “Rhinitis, Allergic, perennial”, “prebiotics”,” probiotics”) and free words (“Seasonal Allergic Rhinitis”, “Pollen Allergy” “Pollinosis”, “Hay Fever”, “allergic rhinitis”, “Perennial Allergic Rhinitis”, “prebiotics”,” probiotics”) to search in the databases aforementioned. The search strategy was as follows, taking PubMed as an example:

(1) (Seasonal Allergic Rhinitis [MeSH Terms]) OR (Perennial Allergic Rhinitis [MeSH Terms]) OR (Allergic Rhinitides, Seasonal) OR (Allergic Rhinitis, Seasonal) OR (Rhinitides, Seasonal Allergic) OR (Rhinitis, Seasonal Allergic) OR (Seasonal Allergic Rhinitides) OR (Pollen Allergy)) OR (Allergies, Pollen) OR (Allergy, Pollen)) OR (Pollen Allergies) OR (Pollinosis)) OR (Pollinoses) OR (Hay Fever)) OR (Fever, Hay) OR (Perennial Allergic Rhinitis) OR (Allergic Rhinitis, Perennial).
(2) (Probiotics [MeSH Terms]) OR (Prebiotics [MeSH Terms]) OR(Probiotics) OR (Prebiotics).
(3) (1) AND (2).

### Eligible Criteria

Studies were included if they met all of the following criteria (1): study design: experimental (randomized and quasi-randomized controlled trials) studies (2); study participants: participants with AR (3); intervention: the intervention group/s should receive probiotics supplementation in any dosage, or regimen as decided by the trialists of the respective trials (4); comparator(s)/control: the participants in the comparison group/s might receive a placebo or other drugs (5); if other drugs were used in the treatment group, they must also be used in the control group in the same way; and (6) language: articles published in the English language.

Articles were excluded if they were published in the form of conference abstract, case report, case series, letter to the editor, correspondence, editorial, narrative reviews, systematic reviews, and meta-analyses.

### Study Selection and Data Extraction

Two investigators independently reviewed titles, abstracts, and full-text articles according to the aforementioned inclusion and exclusion criteria. Disagreement was resolved through discussion or a third investigator. The same two investigators extracted the following data from each selected study: literature characteristics (the first author’s name, journal, year of publication, and study design); participant information (age and sample size); intervention information (intervention duration and comparison group components); outcome (AR and related adverse events); and conclusion.

### Risk of Bias Assessment

The risk of bias assessment was conducted through The Cochrane Risk of Bias Tool Version 1 (21) in Review manager 5.3.4 software by CL and ML. Any disagreement was settled through consultation with the author SP.

### Statistical Analyses

Statistical analyses were completed using Review Manager 5.3.4 software (RevMan; Version 5.3.4. Copenhagen, Denmark: The Nordic Cochrane Centre, The Cochrane Collaboration, 2014). We chose the mean difference (MD) and standardized mean difference (SMD) for continuous outcomes. MD is the difference between the two means, which eliminates the influence of the absolute value between multiple studies. SMD can be simply understood as the quotient of the difference between the two means divided by the combined standard deviation, which not only eliminates the influence of the absolute value of multiple studies, but also eliminates the different effects of multiple study measurement units. Statistical heterogeneity was judged using the inconsistency index (*I* ^2^), and significant heterogeneity was reported if the *I* ^2^ is over 50%. The fixed-effect model was be used in this meta-analysis because larger sample studies will receive greater weight and provide greater contributions to pooled effects. Subgroup analyses were conducted to explore the source of heterogeneity. Publication bias assessment was conducted through funnel plots if more than 10 trials were included. Sensitivity analysis was used to explore the stability of the results. The Grading of Recommendations Assessment, Development and Evaluation (GRADE) Working Group was used to assess the evidence quality for outcomes across studies.

## Results

### Database Search Results

**Figure 1:**
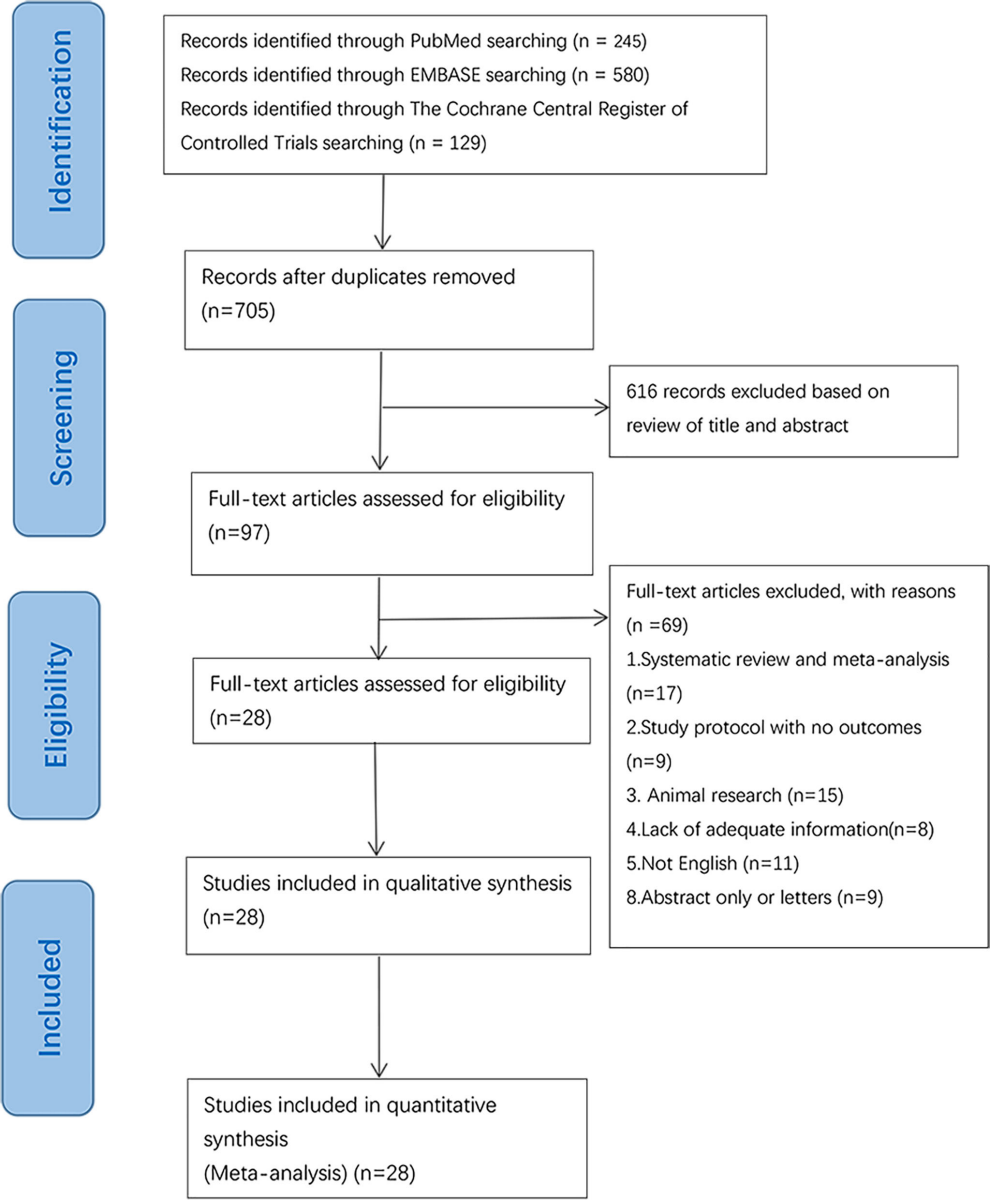
Flowchart of database searching and study identification.

The initial search was completed on June 1, 2021. We have identified 245 potentially relevant publications from PubMed, 580 from Embase, and 129 from The Cochrane Central Register of Controlled Trials. Endnote was used to eliminate duplicate publications, resulting in 97 records for review. After excluding publications that did not meet the inclusion or the exclusion criteria, we included 28 studies for systematic review and meta-Analysis. A flow diagram illustrating the exclusion of articles with specific reasons is shown in **Figure 1** (PRISMA flowchart).

### Study Characteristics

Twenty-eight trials were included in the systematic analysis and meta-analysis. The main characteristics of the individual studies are shown in **Table 1**. Overall, one of these RCTs was a multicenter study (42). Twenty-eight studies included patients from 2 to 65 years of age. Fifteen studies included adults (age > 18 years old) (15–17, 25, 27, 28, 30, 32, 33, 36, 37, 40–42, 44), and eleven studies included children or teenagers (age < 18 years old) (18, 20, 23, 24, 26, 29, 34, 35, 38, 39, 43), and two studies included adults and children (22, 31). Fourteen studies included patients with seasonal allergic rhinitis (SAR) (15–18, 22, 25, 27, 29, 32, 33, 37, 39, 40, 44). Eleven studies included patients with perennial allergic rhinitis (PAR) (20, 23, 24, 28, 31, 32, 34, 36, 38, 41, 42) and three studies included patients with SAR and PAR (26, 30, 43). The intervention group of fourteen studies used *Lactobacillus* strains (17, 20, 22–24, 26–28, 30, 32, 33, 35, 37, 44), and four studies used *Bifidobacterium* strains (16, 25, 36, 39). Three studies used both *Bifidobacterium* strains and *Lactobacillus* strains (18, 40, 42). The other three studies used *Tetragenococcus halophilus* Th22 (31), *E. coli* Nissle 1917 (15), and Broncho-Vaxom (41), respectively. Three studies used probiotics combined with antihistamines (29, 34, 38). One study used *Bifidobacterium* strains and *Enterococcus faecium* (43). The treatment time of probiotics ranged from 6 weeks to 6 months.

**Table 1:** Study characteristics.

Study	Country	Type	Sample size	Participator characteristics	Type of allergic rhinitis	Intervention	Control	Intake of intervention from/until	Outcome	Conclusions	Adverse events/side effects
Helin et al. (22)	Finland	RCT	38	Young adults and teenagers (age 14–36 years old) allergic to birch pollen	Seasonal (birch pollen)	*Lactobacillus rhamnosus* (at least 5×10^9^CFUs/capsule) (2 capsules twice a day)	Placebo (microcrystalline cellulose)(2 capsules twice a day)	5.5 months	1. RTSS (nasal, eye, lung, any symptom) medication use 2. Oral apple challenge	No indication of a beneficial treatment effect in this study	Not mentioned whether any adverse events occurred
Wang et al. (23)	China	RCT	80	Patients(age < 18 years old, mean 15.4 years) had been diagnosed as having perennial allergic rhinitis for more than 1 year	Perennial (Dp)	*Lactobacillus paracasei*-33 (LP-33) (1×10^7^ CFUs/ml) (yogurt/200 ml/day)	Placebo (yogurt) (200 ml/day)	30 days	1. Modified PRQLQ	LP-33-fortified fermented milk can effectively and safely improve the quality of life of patients with allergic rhinitis	No obvious adverse events were found
Peng et al. (24)	China	RCT	90	Children (age > 5 years) old, mean, 15.7 years) with perennial allergic rhinitis characterized by intermittent or continuous nasal symptoms for more than 1 year	Perennial (Dp)	Live or heat-killed *Lactobacillus paracasei* (LP-33) (5×10^9^ CFUs/capsule) two capsules per day	Placebo (two capsules per day)	30 days	1. Modified PRQLQ	1. Heat-killed LP-33 can effectively improve the overall quality of life; 2. The efficacy of the heat-killed LP33 was not inferior to the live variant	No obvious adverse events were found
Xiao et al (16)	Japan	RCT	40	Adult volunteers (age 22–61 years old) with a clinical history of Japanese cedar pollinosis	Seasonal (JCP)	Yogurt with *Bifidobacterium longum* BB536 (approximately 5×10^10^ CFUs/2 g) twice daily	Placebo (yogurt) twice daily	18 weeks	1. Nasal, eye, and throat symptom score, eye drops, and mask wearing 2. Blood sample for total IgE, JCP-specific IgE, IFN-γ, IL-10, or eosinophil rate	BB536-supplementation may relieve JCPsis symptoms	No obvious adverse events were found
Xiao et al. (25)	Japan	RCT	44	Adult volunteers (age 22 57 years old) with a clinical history of Japanese cedar pollinosis	Seasonal (JCP)	Yogurt with *Bifidobacterium longum* BB536 powder [approximately 5× 10^10^ colony-forming units (CFUs)/2 g] twice daily	Placebo (yogurt) twice daily	13 weeks	1. Symptom scores for sneezing, rhinorrhea, nasal blockage, nasal itching, eye, and throat 2. Blood sample for total IgE, JCP-specific IgE, IFN-γ, IL-10, or eosinophil rate	The efficacy of BB536 in relieving JCPsis symptoms through the modulation of Th2-skewed immune response	No obvious adverse events were found
Giovannini et al. (26)	France	RCT	187	Children (age 2–5 years old) with allergic rhinitis or asthma	Perennial and seasonal	Fermented milk containing *Lactobacillus casei* (LcS) (1×10^8^ cfu/ml) 100 ml/day	Placebo (milk) (100 ml/day)	12 months	1. The time free from episodes of asthma/rhinitis 2. Total serum IgA, IgE, IgG, and IgM	Long-term consumption of fermented milk containing *Lactobacillus casei* may improve the health status of children with allergic rhinitis	Abdominal symptoms, diarrhea, and fever episodes
Tamura et al. (27)	Japan	RCT	120	Adults (age >18 years old, mean, 39 years) with allergic rhinitis	Seasonal (JCP)	Fermented milk with *Lactobacillus casei* strain Shirota (LcS)(4×10^10^ CFU/80 ml/day);	Placebo (fermented milk) (80 ml/day)	8 weeks	1. Symptom-medication score, medical (SEM) examination of nasal cavity 2. Blood examination (anti-JCP IgE; eosinophil number; Th1/Th2 relative ratio)	Fermented milk containing LcS does not prevent allergic symptoms in patients sensitive to JCP	No obvious adverse events were found
Ishida et al. (28)	Japan	RCT	52	Adults (age >18 years old, mean, 35.4 years) with perennial allergic rhinitis and high concentrations of anti-house dust IgE or anti house dust mite IgE	Perennial (house dust and mite)	Acidified milk with *Lactobacillus acidophilus* strain L-92 (L-92) (3 × 10^10^ counts/100 ml/day	Placebo (acidified milk) (100 ml/day)	8 weeks	1. Symptom-medication score (SMS) (nasal, ocular) 2. Score of nasal cavity findings 3. Blood sample (total IgE and sIgE levels, Th1/Th2 ratio in blood, eosinophils)	L-92 can alleviate the symptoms of perennial allergic rhinitis	No obvious adverse events were found
Ciprandi et al. (29)	Italy	RCT	20	Children (age 12–15 years old, mean 13.4 years) with allergic rhinitis	Seasonal	*Bacillus clausii* at the dosage schedule of three vials+ levocetirizine (5 mg/day)	Levocetirizine (5 mg/day)	3 weeks	1. Total nasal symptom scores (TNSS) 2. Medication use	*B. clausii* may exert a modulatory effect on allergic response as documented by reduced eosinophil infiltration	Not mentioned whether any adverse events occurred
Ivory et al. (30)	England	RCT	20	AR sufferers (age 18–45 years old) with a history of seasonal allergic rhinoconjuctivitis	Perennial and seasonal	Probiotic drinks contain *Lactobacillus casei* Shirota (LcS) (6.5 ×10^9^ LcS/65 ml/day)	Placebo (placebo drinks/65ml/day)	5 months	1. Blood examination (IL-1b, IL-2, IL-4, IL-5, IL-6, IL-8, IL-10, IL-12p70, IFN-g, and TNF-a)	Probiotic supplementation modulates immune responses in allergic rhinitis	Not mentioned whether any adverse events occurred
Nishimura et al. (31)	Japan	RCT-DB	45	Subjects (age 16–60 years old) with perennial allergic rhinitis and had a history of PAR of more than 3 years	Perennial (house dust or mites)	*Tetragenococcus halophilus* Th22 (high-dose tablets that contain 10 mg/tablet, 6 tablets/day; low-dose tablets that contain 3.4 mg/tablet, 6 tablets/day)	Placebo (6 tables/day)	8 weeks	1. Total nasal symptom scores (TNSS) (combination of sneezing, rhinorrhea, and nasal obstruction) 2. Serum total IgE and sIgE levels, eosinophil count, nasal eosinophil, and neutrophil counts	Th221 can be expected to safely improve the symptoms of PAR	No obvious adverse events were found
Kawase et al. (32)	Japan	RCT	40	Adults (age >18 years old, mean, 36.9 years) with a clinical history of Japanese cedar pollinosis	Seasonal (JCP)	Fermented milk contains usual bacteria and *Lactobacillus* GG and *L. gasseri* TMC0356 (110 g/day)	Placebo (yogurt contains the usual bacteria) (110 g/day)	10 weeks	1. Symptom score (sneezing, rhinorrhea, itching) 2. Symptom-medication score 3. Blood examination (total IgE, sIgE, Th1/Th2 ratio, TARC, CRP, eosinophils)	The fermented milk prepared with LGG and TMC0356 might be beneficial in JCP	Not mentioned whether any adverse events occurred
Ouweh et al. (18)	Sweden	RCT	47	Children (age 4–13 years old) with clinically and immunologically documented and physician-verified birch pollen allergy	Seasonal(birch pollen);	A combination of *Lactobacillus acidophilus* and *Bifidobacterium lactis* (5x10^9^ CFU/capsules/day)	Placebo (one capsule/day)	4 months	1. Presence of nasal, respiratory, or ocular symptoms; 2. Serum sIgE level, blood 3. Nasal eosinophil counts, cytokines IL-4, IL-5, IL-6, IL-10, TNF-α, TGF-β2, soluble CD14 4. Fecal microbiota, calprotectin, and IgA	1. Probiotics prevent the infiltration of eosinophils into the nasal mucosa; 2. Probiotics reduce nasal symptoms	Not mentioned whether any adverse events occurred
Yonekura et al. (17)	Japan	RCT	126	Patients (age 20–50 years old) with Japanese cedar pollinosis	Seasonal (JCP)	*Lactobacillus paracasei* strain KW3110 (1×10^12^–3×10^12^ CFU/g/day)	Placebo (dextrin) (1 g/day)	3 months	1. Nasal symptoms (sneezing, runny nose, stuffy nose) 2. Quality-of-life score 3. Blood examination (total IgE, sIgE, serum eosinophil count and ECP, Th1/Th2 ratio);	1. KW3110 can significantly reduce nasal symptoms and the serum level of eosinophil cationic protein 2. KW3110 can improve quality-of-life scores when pollen scattering was low	Loose stools; diarrhea
Nagata et al. (33)	Japan	RCT-DB	35	Female college students (age 18–27 years old) with seasonal allergic diseases	Seasonal (JCP)	*Lactobacillus plantarum* No. 14 (LP14) (8.7 ×10^8^CFU/0.5 g) (0.5 g/day)	Placebo (branched dextrin) (0.5 g/day)	6 weeks	1. Scores for ocular SME, itchy eyes, and medicine taking 2. Total IgE, anti-JCP IgE, eosinophil count, CRP; and Th1 percentage, Th2 percentage, and Th1/Th2 ratio, antiragweed, anti-house dust mite IgE, fecal microbiota	LP14 strongly induced the gene expression of Th1-type cytokines, which indicates the clinical effects of LP14 on seasonal allergic rhinitis	No obvious adverse events were found
Jan et al. (20)	China	RCT-DB	240	Patients (age < 18 years old, mean: 8 years) with history of perennial allergic symptoms for at least 3 years	Perennial (Dp, Df, or dust)	*Lactobacillus rhamnosus* (4×10^9^ CFU/g) (1 g/day)	Placebo (microcrystalline cellulose) (1 g/day)	12 weeks	1. Nasal, eye, lung symptom clinical score 2. Blood cell counts, total IgE, and blood eosinophil counts	*L. rhamnosus* treatment neither reduced rhinitis symptom scores nor altered immunological parameters in symptomatic children	Not mentioned whether any adverse events occurred
Lue et al. (34)	Sweden	RCT	63	Children (age 7–12 years old) with moderate-to-severe perennial allergic rhinitis	Perennial(house dust mite);	Levocetirizine (5 mg/day)with *Lactobacillus johnsonii* EM1 (Lj EM1) (1×10^10^ CFU/capsule/day)	Levocetirizine (5 mg/day)	12 weeks	1. Daily diary of total symptom score and sleep quality 2. The Pediatric Rhinoconjunctivitis Quality of Life (PRQLQ) 3. Nasal peak expiratory flow rate 4. Nasal smear 5. Peripheral blood eosinophils, total serum IgE, mite-specific IgE, ECP, resistin, IL4, IL-10, IFN-g, and TGF-b	Levocetirizine plus Lj EM1 was more effective for perennial allergic rhinitis than levocetirizine and that this difference persisted for at least 3 months after discontinuation of Lj EM1	No obvious adverse events were found
Lin et al. (35)	Sweden	RCT-DB	199	Children (6–12 years old) have a history of perennial allergic symptoms for at least 3 years	Perennial (Dp, Df, or dust)	*Lactobacillus salivarius* PM-A0006 (4×10^9^ CFUs/g) (500 mg/day)	Placebo (500 mg/day)	12 weeks	1. Specific symptom scores for eye, nose, lung, medicine 2. Eosinophil count, total IgE level	*Lactobacillus salivarius* treatment reduces rhinitis symptoms and drug usage in children with allergic rhinitis	Not mentioned whether any adverse events occurred
Singh et al. (36)	Switzerland	RCT-DB	20	Adult subjects (age 20–65 years old) with clinical history of SAR and positive skin prick test to grass pollen	Perennial (house dust and mite)	*Bifidobacterium lactis* NCC2818 (2×10^9^CFU/day) 2 g/day	Placebo (2 g/day)	8 weeks	1. TNSS 2. IL-2, IL-5, IL-10, IFN-γ, IL-13, IL-1, and TNF-1β in whole-blood cell cultures; total IgE and sIgE level	Oral administration of the probiotic NCC2818 mitigates immune parameters and allergic symptoms during seasonal exposure	No obvious adverse events were found
Dölle et al. (15)	Germany	RCT-DB	34	Subjects (age 18–65 years old) with grass pollen-dependent allergic rhinoconjunctivitis	Seasonal (JCP)	2.5–25 billion viable bacteria of the strain *E. coli* Nissle 1917 (1 capsule daily over the first 4 days, 2 capsules daily until the end of treatment)	Placebo (1 capsule daily over the first 4 days, 2 capsules daily until the end of treatment)	6 months	1. SMS during grass-pollen season 2. Skin-prick test, conjunctival provocation test, RQLQ, total IgE, sIgE, sIgA levels	6 months of coseasonal nonspecific immunomodulation by EcN is not sufficient to achieve clinical efficacy in grass pollen-allergic subjects	Diarrhea, abdominal pain, flatulence
Costa et al. (37)	France	RCT-DB	425	Subjects (age 18–60 years old) with persistent AR, symptomatic during the grass pollen season, and a positive skin test or specific immunoglobulin E to grass pollens	Seasonal (grass)	*Lactobacillus paracasei* subsp. (paracasei LP-33) 2.0×10^9^ CFU/capsule/day + loratadine (10 mg/day)	Placebo (one capsule/day) + loratadine(10 mg/day)	5 weeks	1. The RQLQ global score 2. Nasal and ocular symptoms	LP-33 improves the quality of life of subjects with persistent AR who are currently being treated with an oral H1-antihistamine. Whereas nasal symptoms had not changed, ocular symptoms had consistently improved	No obvious adverse events were found
Lin et al. (38)	China	RCT	60	Children (age 6–13 years old) had perennial allergic rhinitis for more than 1 year	Perennial (house dust mites);	Levocetirizine (8 weeks) +*Lactobacillus paracasei* (HF.A00232) (4 weeks);	Levocetirizine (8 weeks) +placebo (4 weeks)	12 weeks	1. PRQLQ 2. sIgE, IL-4, IFN-γ, IL-10, TGF-β	Dietary supplementation with LP (HF.A00232) provided no additional benefit when used with regular levocetirizine in treating AR in the initial 8 weeks, but there was a significant improvement in individual symptoms of sneezing, itchy nose, and swollen eyes, after discontinuing regular levocetirizine treatment	No obvious adverse events were found
Nembrini et al. (19)	England	RCT-DB	131	Grass pollen allergic subjects (age 18–65 years old)	Seasonal (grass pollen)	A probiotic blend containing 5 × 10^9^ CFU *Lactobacillus paracasei* NCC 2461 (5 g/day)	Placebo (maltodextrin);(5 g/day)	8 weeks	1. TNSS 2. RQLQ 3. Medication score	Oral administration of NCC 2461 did not show a beneficial effect on allergic rhinitis	No obvious adverse events were found
Delgiudice et al. (39)	Italy	RCT-DB	40	Patients (age 4–17 years old) with allergic rhinitis and intermittent asthma due to *Parietaria officinalis* pollen	Seasonal (*Parietaria officinalis* pollen)	A mixture powder composed of three bifidobacteria *Bifidobacterium Longum* BB536 (3 billion units) + *Bifidobacterium infantis* M-63 (1 billion units) + *Bifidobacterium breve* M-16 V (1 billion units) (0.5 ml per os all days for 2 months)	Placebo (0.5 ml per os all days for 2 months)	2 months	1. RTSS 2. Quality of life (QoL)	A bifidobacteria mixture was capable of significantly improving AR symptoms and QoL in children with pollen-induced AR and intermittent asthma	No obvious adverse events were found
Dennis-wall et al. (40)	America	RCT-DB	173	Participants (age 18–60 years old) who typically receive a global score of ≥2 on the MRQLQ during peak allergy season	Seasonal	*Lactobacillus gasseri* KS-13, *Bifidobacterium bifidum* G9-1, and *B. longum* MM-2 (1.5 billion CFU/capsule) (2 capsules/day, 1.5 billion colony-forming units/capsule)	Placebo (348 mg potato starch) twice a day	8 weeks	1. Rhinoconjunctivitis-specific quality of life (MRQLQ) 2. Gastrointestinal function 3. Immune markers	Probiotic improved rhinoconjunctivitis-specific quality of life during allergy season for healthy individuals with self-reported seasonal allergies	No obvious adverse events were found
Meng et al. (41)	China	RCT-DB	60	Patients (age > 18 years, mean, 31.34 years) with moderate to severe perennial AR for >2 years	Perennial	Broncho-Vaxom (BV) (7 mg/day);	Placebo (7 mg/day)	3 cycles (10 consecutive days followed by a 20-day resting period/cycle)	1. Individual nasal symptom score (INSS) 2. Total nasal symptom score (TNSS) 3. IL-4, IL-13, and interferon (IFN)-γ	Oral administration of BV may be considered as an alternative therapeutic strategy for patients with persistent AR	Slight abdominal pain (adverse events were spontaneously alleviated without drug treatment)
Kang et al. (42)	South Korea	Multicenter randomized controlled study	95	Subjects (age 19–65 years old) with persistent rhinitis symptoms for at least two consecutive years	Perennial (Dp, Df, cat, dog, and cockroach)	Probiotic NVP-1703 (a mixture of *Bifidobacterium longum* and *Lactobacillus plantarum*) [1.0 × 10^10^ CFU/day (2 g/stick pack)]	Placebo (maltodextrin) (2 g/stick pack)	4 weeks	1. TNSS(nasal congestion, rhinorrhea, nasal itching, and sneezing) 2. RCAT 3. Blood eosinophil count 4. Allergen-specific IgE, and immunological parameters in serum (IL-4, IL-5, IL-10, IL-13, IFN-γ);	NVP-1703 can be treatment option for perennial AR	No obvious adverse events were found
Anania et al. (43)	Italy	RCT-DB	250	Children (age 6–17 years) with allergic rhinitis, undergoing treatment with conventional AR therapies [antihistamines (oral)+corticosteroids (local)]	Perennial (dust);and seasonal (grass pollen)	*Bifidobacterium animalis* subsp. Lactis BB12 and *Enterococcus faecium* L3 (2 × 10^9^ CFUs/2.5 g/ sachet) (one sachet per day)	Placebo (maltodextrin) (one sachet per day)	3 months	1. Nasal symptoms score 2. Pharmacological treatment of AR	A mixture of BB12 and L3 statistically decreased signs and symptoms of AR and reduced significantly the need of conventional therapy	No obvious adverse events were found

Total nasal symptom scores (TNSS), rhinoconjunctivitis total symptom score (RTSS), rhinitis control assessment test (RCAT), Mini Rhinoconjunctivitis Quality of Life Questionnaire (MRQLQ), colony-forming units (CFUs), Dermatophagoides pteronyssinus (Dp), and Dermatophagoides farinae (Df).

### Risk of Bias Assessment

**Figure 2:**
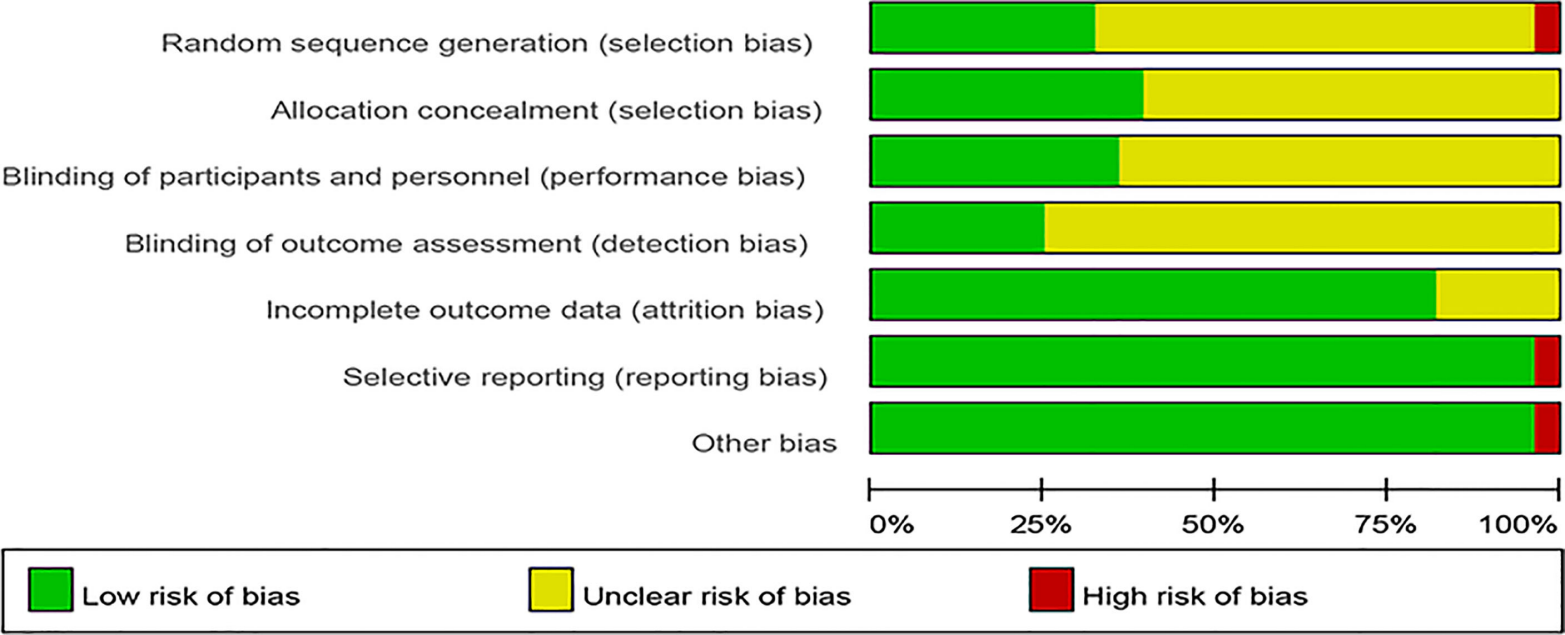
Risk of bias.

**Figure 3:**
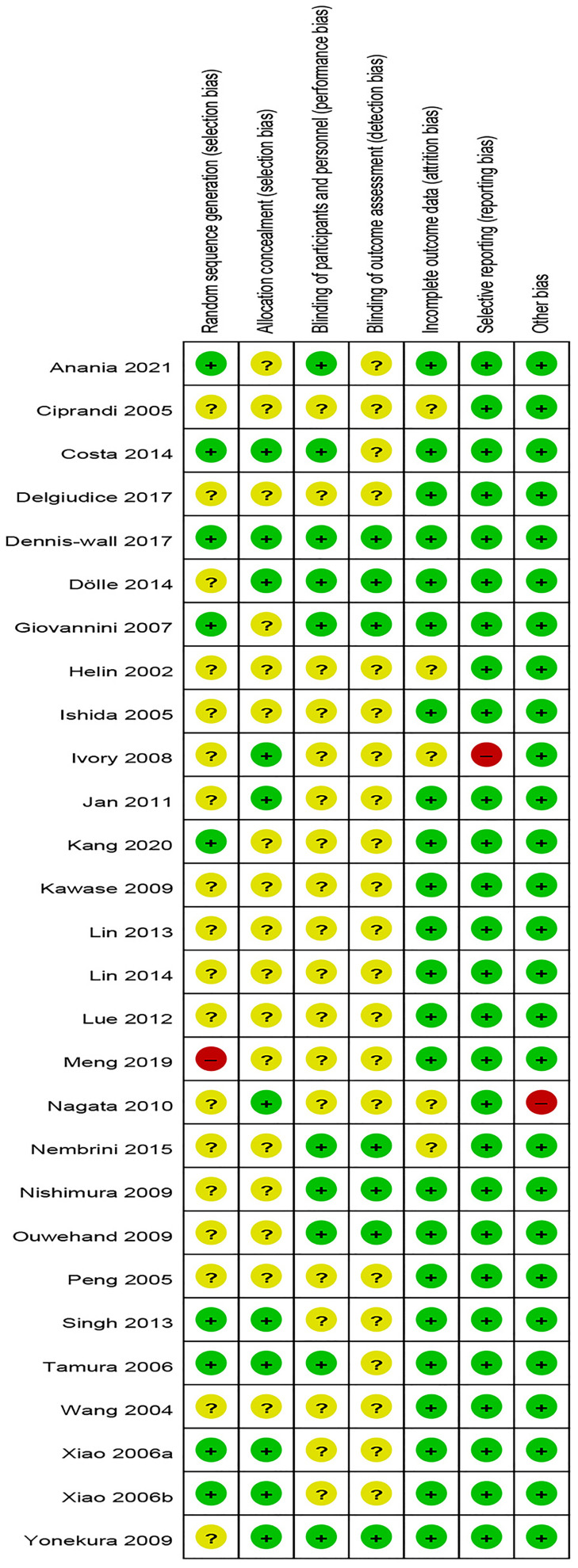
Summary of risk of bias.

The risk of bias assessment is presented in **Figures 2**, **3**. Most studies did not clearly show how to generate random sequences, nor did they clearly state whether association obfuscation was performed. In terms of masking method, most of the studies have insufficient information to permit judgment of “Low risk” or “High risk”. We assessed three trials having high risk of bias for different reasons. One of the trials did not report all the pre-specified primary outcome indicators (30). The random allocation method in one of the studies was incorrect (The patients were randomized according to the birth date) (41). Since Nagata reported that participants were all female college students from the same university in the trial (33), it was marked as “high risk” in other bias.

### Overall Analyses

#### Allergic Rhinitis Symptoms Score

**Figure 4:**
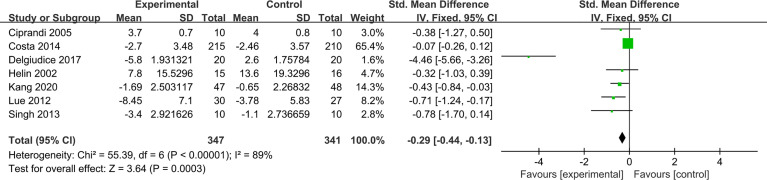
Forest plot for allergic rhinitis symptoms score.

AR symptoms score included rhinoconjunctivitis total symptom score (RTSS) and total nasal symptom scores (TNSS). RTSS includes five individual AR symptoms (nasal congestion, sneezing, rhinorrhea, nasal pruritus, and eye itching) noted from 0 (no symptom) to 3 (severe symptom). TNSS were expressed as the sum of the scores for the four symptoms (nasal congestion, rhinorrhea, nasal itching, and sneezing) noted from 0 (no symptom) to 3 (severe symptom). Seven trials reported pre- and post-treatment data of AR symptoms score available for meta-analysis. Compared with placebo, probiotics significantly improved AR symptoms score (SMD, −0.29, 95% CI [−0.44, −0.13]). There was high heterogeneity in the result (*p* = 0.0003, *I* ^2^ = 89%) (**Figure 4**). Sensitivity analysis indicates that the result is robust (**Supplementary Material 13**). Due to the significantly statistical heterogeneity encountered in the analysis, several subgroup analyses were conducted separately according to the classification of AR, combination of drugs, and intervention of treatment group.

With regard to classification of AR, probiotics can significantly relieve symptoms in patients with SAR (SMD −0.56, 95% CI [−0.87, −0.25]; *p* = 0.0003, *I* ^2^ = 0%), and there was significant benefit that probiotics supplementation relieved PAR symptoms score (SMD,−0.19, 95% CI [−0.37, −0.01]; *p* = 0.03, *I* ^2^ = 94%) (**Supplementary Material 1**). Subgroup analysis according to the combination of drugs again found some evidence of a protective effect of probiotics (monotherapy) in relieving AR symptoms compared with placebo (SMD, −0.73, 95% CI [−1.05, −0.42]; *p* < 0.00001, *I* ^2^ = 93%). Compared with antihistamines, probiotics combined with antihistamines (combination therapy) have no significant relief of AR symptoms (SMD, −0.15, 95% CI [−0.32, 0.03]; *p* = 0.10, *I* ^2^ = 61%) (**Supplementary Material 2**). The results of subgroup analysis showed that probiotics (single) compared with placebo cannot significantly relieve symptoms (SMD, −0.49, 95% CI [−1.05, 0.07], *p* = 0.09). Similarly, probiotics combined with antihistamines compared with antihistamines have no significant relief of AR symptoms (SMD, −0.15, 95% CI [−0.32, 0.03], *p* = 0.10, *I* ^2^ = 61%). Probiotics (mixed) compared with placebo have significant relief of AR symptoms (SMD, −0.85, 95% CI [−1.23, −0.46], *p* < 0.0001, *I* ^2^ = 97%) (**Supplementary Material 3**) (**Table 2**).

**Table 2:** Subgroup analysis for outcomes.

	Number of comparisons	Results	*p*-value for overall effect	*I* ^2^	*p-*value for subgroup difference
		**Std. Mean Difference (95%)**			
**Allergic Rhinitis Symptoms Score**					
All comparisons	7	−0.29 [−0.44, −0.13]	*p* = 0.0003	89%	
**Classification of allergic rhinitis**					*p* = 0.04
Perennial allergic rhinitis (PAR)	4	−0.19 [−0.37, −0.01]	*p* = 0.03	94%	
Seasonal allergic rhinitis (SAR)	3	−0.56 [−0.87, −0.25]	*p* = 0.0003	0%	
**Combination of drugs**					*p* = 0.02
Monotherapy	4	−0.73 [−1.05, −0.42]	*p* < 0.00001	93%	
Combined (probiotics combined with antihistamines)	3	−0.15 [−0.32, 0.03]	*p* = 0.10	61%	
**Intervention of treatment group**					*p* = 0.004
Probiotics combined with antihistamines	3	−0.15 [−0.32, 0.03]	*p* = 0.10	61%	
Mixed probiotics	2	−0.85 [−1.23, 0.46]	*p* < 0.0001	97%	
Single probiotic	2	−0.49 [−1.05, −0.07]	*p* = 0.09	0%	
		**Std. Mean Difference (95%)**			
**Rhino-conjunctivitis Quality of Life Questionnaire Score**					
All comparisons	7	−0.64 [−0.79, −0.49]	*p* < 0.00001	97%	
**Classification of allergic rhinitis**					*p* < 0.00001
Perennial allergic rhinitis (PAR)	4	−2.10 [−2.45, −1.74]	*p* < 0.00001	97%	
Seasonal allergic rhinitis (SAR)	3	−0.32 [−0.49, −0.15]	*p* = 0.0002	96%	
**Combination of drugs**					*p* < 0.00001
Monotherapy (probiotics)	5	−1.74 [−2.03, −1.46]	*p* < 0.00001	97%	
Combined (probiotics combined with antihistamines)	2	−0.21 [−0.39, −0.03]	*p* = 0.02	0%	
**Intervention of treatment group**					*p* < 0.00001
Probiotics combined with antihistamines	2	−0.21 [−0.39, −0.03]	*p* = 0.02	0%	
Mixed probiotics	1	−5.16 [−6.50, −3.81]	*p* < 0.00001	NA	
Single probiotic (IL-33)	3	−3.81 [−4.29, −3.32]	*p* < 0.00001	0%	
		**Std. Mean Difference (95%)**			
**Total IgE**					
All comparisons	9	−0.03 [−0.18, 0.13]	*p* = 0.72	0%	
**Classification of allergic rhinitis**					0.34
Perennial allergic rhinitis and Seasonal allergic rhinitis (PAR and SAR);	1	−0.19 [0.48, 0.10]	–	NA	
Perennial allergic rhinitis(PAR);	5	0.07 [−0.13, 0.27]	*p* = 0.50	8%	
Seasonal allergic rhinitis (SAR)	3	−0.09 [0.48, 0.30]	*p* = 0.65	0%	
**Combination of drugs**					*p* = 0.82
Monotherapy (probiotics)	8	−0.03 [−0.19, 0.13]	*p* = 0.69	0%	
Combined (probiotics combined with antihistamines)	1	0.03 [−0.49, 0.55]	–	NA	
		**Std. Mean Difference (95%)**			
**sIgE**					
All comparisons	6	0.09 [−0.16, 0.34]	*p* = 0.49	0%	
**Classification of allergic rhinitis**					0.40
Perennial allergic rhinitis (PAR)	2	−0.03 [−0.41, 0.34]	*p* = 0.86	0%	
Seasonal allergic rhinitis (SAR)	4	0.18 [−0.15, 0.51]	*p* = 0.28	0%	
**Combination of drugs**					0.12
Monotherapy (probiotics)	5	0.00 [−0.27, 0.27]	*p* = 0.99	0%	
Combined (probiotics combined with antihistamines)	1	0.55 [−0.08, 1.18]	*p* = 0.09	NA	
		**Mean Difference (95%)**			
**Th1/Th2 ratio**					
All comparisons	4	−2.01 [−3.94, −0.08]	*p* = 0.04	72%	
**Classification of allergic rhinitis**					*p* = 0.02
Perennial allergic rhinitis (PAR)	1	−1.50 [−2.63, −0.37]	*p* = 0.01	NA	
Seasonal allergic rhinitis (SAR)	3	−3.42 [−4.54, −2.30]	*p* = 0.04	72%	
**Combination of drugs**					*p* = 0.002
Monotherapy (probiotics)	3	−1.34 [−2.41, −0.28]	*p* = 0.01	0%	
Combined (probiotics combined with antihistamines)	1	−3.9 [−5.10, −2.70]	*p* < 0.00001	NA	

NA, not applicable.

#### Rhinoconjunctivitis Quality of Life Questionnaire Score

**Figure 5:**
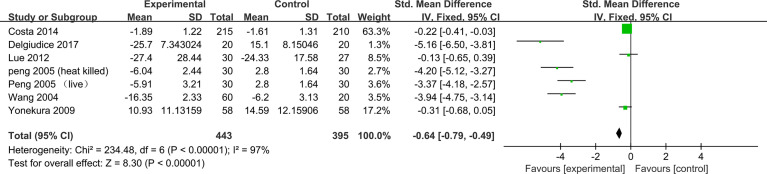
Forest plot for Rhinoconjunctivitis Quality of Life Questionnaire Score.

Seven trials reported pre- and post-treatment data of Rhinoconjunctivitis Quality of Life Questionnaire (RQLQ) scores available for meta-analysis. The results combined with the fixed-effect model showed a significant decrease in RQLQ scores in the probiotic group compared with the control group (−0.64, 95% CI [−0.79, −0.49], *p* < 0.00001, *I* ^2^ = 97%) (**Figure 5**). Sensitivity analysis indicates that the result is stable (**Supplementary Material 13**).

Subgroup analysis according to the classification of AR found some evidence of a significant decrease in RQLQ scores for SAR in the probiotic group compared with the control group (SMD, −0.32, 95% CI [−0.49, −0.15], *p* = 0.0002, *I* ^2^ = 96%), and a greater beneficial effect in PAR (SMD, −2.10, 95% CI [-2.45, −1.74], *p* < 0.00001, *I* ^2^ = 97%) (**Supplementary Material 4**). Subgroup analysis according to the combination of drugs again found some evidence of a protective effect of probiotics (monotherapy) in relieving AR symptoms compared with placebo (SMD, −1.74, 95% CI [−2.03, −1.46]; *p* < 0.00001, *I* ^2^ = 97%). Compared with antihistamines, probiotics combined with antihistamines (combination therapy) have a significant relief of AR symptoms (SMD, −0.21, 95% CI [−0.39, −0.03]; *p* = 0.02, *I* ^2^ = 0%) (**Supplementary Material 5**). The results of subgroup analysis showed that probiotics (single) comparing with placebo can significantly relieve symptoms (SMD, -3.81,95% CI [-4.29, -3.32], p<0.00001, I2=0%). Similarly, probiotics combined with antihistamines (combination therapy) compared with antihistamines showed significant improvement in RQLQ scores (SMD, −0.21, 95% CI [−0.39, −0.03], *p* = 0.02, *I* ^2^ = 0%) (**Supplementary Material 6**) (**Table 2**).

#### Immunologic Parameters

##### Total IgE

**Figure 6:**
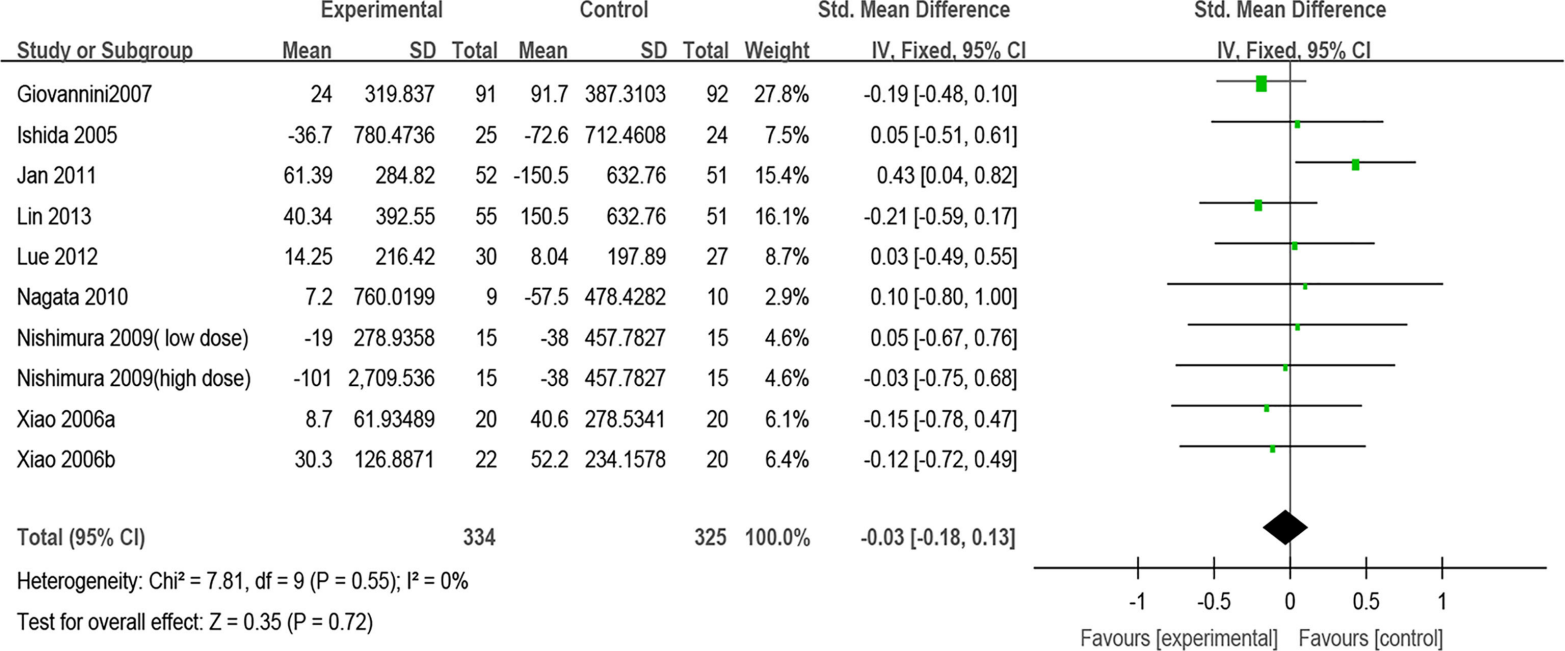
Forest plot for Total IgE.

Nine trials reported the effect of probiotics on total IgE. After pooling nine estimates, there was no difference found in total IgE between the probiotic group and the control group (SMD, −0.03, 95% CI [−0.18, 0.13], *p* = 0.72, *I* ^2^ = 0%) (**Figure 6**). Sensitivity analysis indicates that the result is stable (**Supplementary Material 13**). Subgroup analyses were conducted according to the classification of AR and combination of drugs. The results of subgroup analysis showed that the effect of probiotics on total IgE could not be affected by the classification of AR (PAR or SAR) or combined with other drugs (**Supplementary Materials 7** and **8**) (**Table 2**).

##### Specific IgE

**Figure 7:**
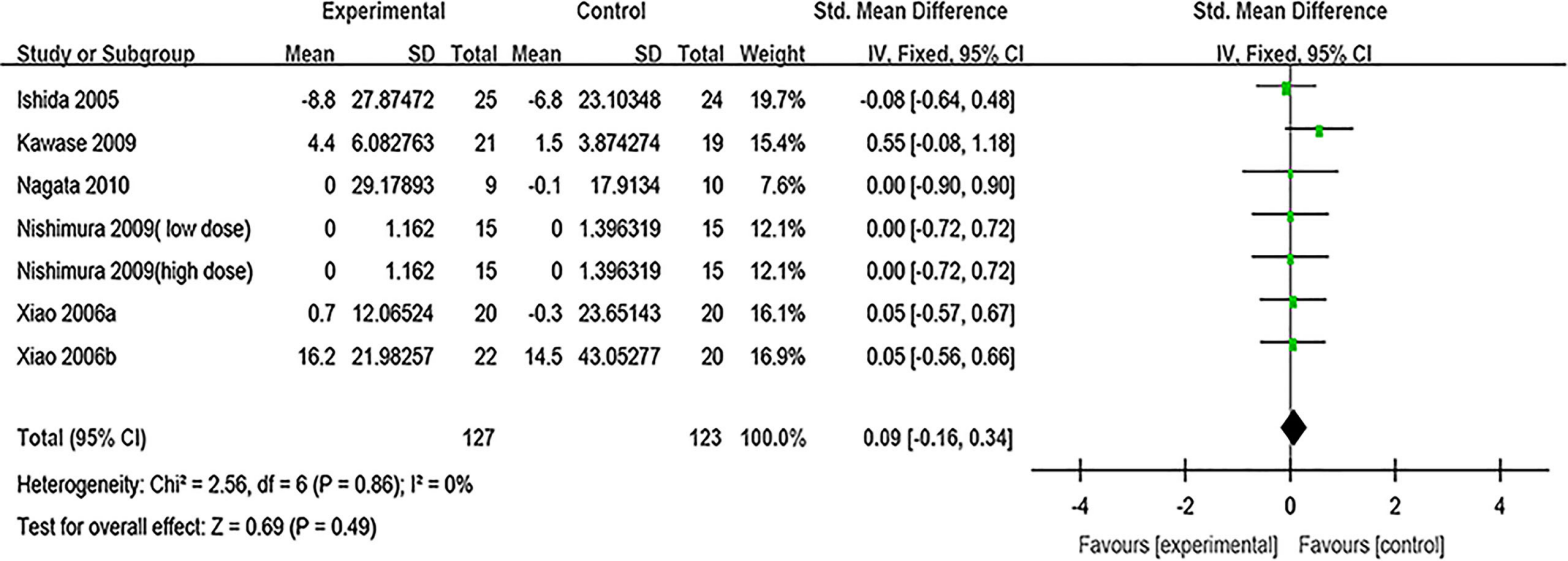
Forest plot for sIgE.

Specific IgE was evaluated in six studies. After pooling six estimates, there was no difference found in sIgE between the probiotic group and the control group (SMD, 0.09, 95% CI [−0.16, 0.34], *p* = 0.49, *I* ^2^ = 0%) (**Figure 7**). Sensitivity analysis indicates that the result is stable (**Supplementary Material 13**). Subgroup analyses were conducted according to the classification of AR and combination of drugs. The results of subgroup analysis showed that the effect of probiotics on sIgE could not be affected by the classification of AR (PAR or SAR) or combined with other drugs (**Supplementary Materials 9** and **10**) (**Table 2**).

##### Th1/Th2 ratio

**Figure 8:**
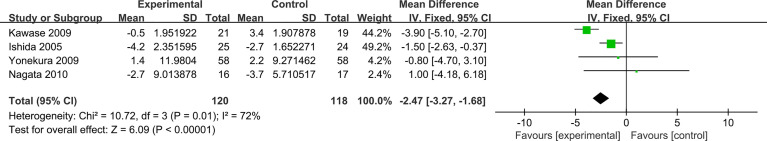
Forest plot for Th1/Th2 ratio.

Four trials reported enough data to allow meta-analysis for the Th1/Th2 ratio. The results showed that the Th1/Th2 ratio was lower in the control group when the effect estimates from four trials were pooled (MD, −2.47, 95% CI [−3.27, −1.68], *p* < 0.00001, *I* ^2^ = 72%) (**Figure 8**). Sensitivity analysis indicates that the result is stable (**Supplementary Material 13**). Subgroup analyses were conducted according to the classification of AR. The results of subgroup analysis showed that the effect of probiotics on the Th1/Th2 ratio could not be affected by the classification of AR (PAR or SAR) or treatment plan (monotherapy/combined) (**Supplementary Materials 11** and **12**) (**Table 2**).

#### Adverse Events

Of the twenty-eight studies included, seventeen RCTs mentioned that no obvious adverse events were found during the research, while seven RCTs did not mention whether any adverse events occurred. Four RCTs have reported adverse events including diarrhea, abdominal pain, flatulence, and fever episodes. One study reported that loose stools and diarrhea were observed in the active and placebo groups, which had no significant differences in adverse events between the two groups (chi-square test, *p* < 0.4) (17). Another study showed that subjects with these adverse drug reactions (diarrhea, abdominal pain, and flatulence) recovered within a few days. In this study, it was found that one subject’s adverse reaction was almost certainly related to the drug (15). One study reported slight abdominal pain in probiotic groups and all of the adverse events were spontaneously alleviated without drug treatment (41). One study revealed that abdominal symptoms (abdominal symptoms, diarrhea, and fever episodes) were reported in 56.5% versus 64.2% of children in intervention and control groups, respectively (*p* = 0.282) (26).

#### GRADE Evidence Quality Evaluation

The quality of evidence applied for each outcome is summarized in **Table 3**. The quality of evidence on the Allergic Rhinitis Symptoms Score, Rhinoconjunctivitis Quality of Life Questionnaire Score, Total IgE, Antigen-specific IgE, and Th1/Th2 ratio was rated as very low, very low, low, low, and very low, respectively (**Table 3**).

**Table 3:** GRADE assessment.

Outcomes	Illustrative comparative risks* (95% CI)		Relative effect (95% CI)	No of participants (studies)	Quality of the evidence (GRADE)
	Assumed risk	Corresponding risk			
	**Control**				
**Allergic Rhinitis Symptoms Score**		The mean RTSS global score in the intervention groups was **0.29 standard deviations lower** (0.44 to 0.13 lower)	SMD −0.29 (−0.44 to −0.13)	688 (7 studies)	⊕⊝⊝⊝ **Very low** ^1,2,3^
**Rhino-conjunctivitis Quality of Life Questionnaire Score**		The mean RQLQ global score in the intervention groups was **2.38 standard deviations lower** (3.58 to 1.19 lower)	SMD −2.38 (−3.58 to −1.19)	838 (7 studies)	⊕⊝⊝⊝ **Very low** ^1,2,3^
**Total IgE**		The mean total IgE in the intervention groups was **0.03 standard deviations lower** (0.18 lower to 0.13 higher)	SMD −0.03 (−0.18 to 0.13)	659 (10 studies)	⊕⊕⊝⊝ **Low** ^1,3^
**Antigen-specific IgE**		The mean antigen-specific IgE in the intervention groups was **0.09 standard deviations higher** (0.16 lower to 0.34 higher)	SMD 0.09 (−0.16 to 0.34)	250 (7 studies)	⊕⊕⊝⊝ **Low** ^1^,^3^
**Th1/Th2**		The mean Th1/Th2 in the intervention groups was **2.47 lower** (3.27 to 1.68 lower)	MD −2.47 [−3.27, −1.68]	238 (4 studies)	⊕⊝⊝⊝ **Very low** ^1,3^

*The basis for the assumed risk (e.g., the median control group risk across studies) is provided in footnotes. The corresponding risk (and its 95% confidence interval) is based on the assumed risk in the comparison group and the relative effect of the intervention (and its 95% CI).

CI, Confidence interval.

GRADE Working Group grades of evidence.

High quality, Further research is very unlikely to change our confidence in the estimate of effect.

Moderate quality, Further research is likely to have an important impact on our confidence in the estimate of effect and may change the estimate.

Low quality, Further research is very likely to have an important impact on our confidence in the estimate of effect and is likely to change the estimate.

Very low quality, We are very uncertain about the estimate.

^1^In some studies, random sequence generation, allocation concealment, and blinding of participants and personnel are not described.

^2^There is a significant heterogeneity (I^2^ > 50%).

^3^PICO is not exactly the same.

## Discussion

In this study, the clinical evidence of probiotics in the treatment of AR was systemically collated and analyzed so as to provide a better guidance for clinical practice. Our results showed that probiotics supplementation for patients with AR can ameliorate AR symptoms and improve the quality of life. Probiotics supplementation can correct the Th1/Th2 balance. There was no significant change in overall or antigen-specific IgE levels between probiotic-treated and placebo-treated subjects. The results of this study have significant heterogeneity, and the source of heterogeneity was explored by subgroup analysis. The results of subgroup analysis showed that probiotics can significantly relieve AR symptoms in patients with SAR. Subgroup analysis according to combination of drugs again found some evidence of a protective effect of probiotics (monotherapy) in relieving AR symptoms compared with placebo. Compared with antihistamines, probiotics combined with antihistamines (combination therapy) have no significant relief of AR symptoms. Subgroup analyses of these outcomes failed to find out the source of heterogeneity. The different doses, durations, and strains of probiotics may be the sources of heterogeneity. With regard to RQLQ score, the results of subgroup analysis according to combination of drugs showed that probiotics (single probiotic strain) compared with placebo can significantly improve the quality of life. Similarly, probiotics combined with antihistamines (combination therapy) compared with antihistamines showed a significant decrease in RQLQ scores, which means an improvement in the quality of life. As we all know, helper T cells play a key role in the adaptive immune response. Human T helper cells can be divided into two main subtypes, Th1 and Th2. The significant trend of immune response to Th2 lineage may lead to allergic diseases. Immunoglobin E (IgE)-mediated allergic inflammation is the main pathophysiological mechanism of AR and drives T helper 2 (Th2) cell polarized immune reactions (45).

The balance Th1/Th2 is associated with AR. Th2 induces the activation of B cells and IgE class switching, which leads to B-cell differentiation into plasma cells that produce allergen-specific IgE. IgE enters the circulation and binds through its Cϵ3 domain to the high-affinity IgE receptor (FcϵRI) on the surface of mast cells and basophils (46). Activated mast cells and basophils release inflammatory mediators (e.g., histamine and leukotrienes) that cause symptoms such as nasal itching, sneezing, and runny nose. At the same time, these inflammatory mediators lead to a predominance of Th2 immune responses, further exacerbating inflammation. Therefore, the predominance of Th2 and its related cytokines correlates with the severity of AR. The Th1/Th2 ratio can reflect the effect of improving allergy symptoms by drugs to a certain degree.

Our meta-analysis demonstrated that probiotics supplementation can correct the Th1/Th2 balance, which indicates that probiotic supplementation can ameliorate AR by regulating the balance of Th1/Th2. However, only four of the included studies reported the Th1/Th2 ratio.

The purpose of most systematic reviews or meta-analyses is to explore the preventive effect of probiotic supplementation on allergic diseases (47–50). There are less systematic reviews or meta-analyses to explore the therapeutic effect of probiotics on AR. A systematic review and meta-analysis of probiotics in the treatment of AR published in 2015 has shown that probiotics may be beneficial in improving symptoms and quality of life in patients with AR (51). One meta-analysis showed that probiotics have beneficial effects in the treatment of AR, especially with SAR and LP-33 strains (52). However, previous systematic reviews failed to explore the causes of heterogeneity as much as possible. Compared with previous systematic reviews and meta-analyses, our meta-analysis conducted subgroup analysis according to types of AR (PAR/SAR) and treatment plan (single probiotic strain/mixed probiotic strains/probiotics combined with antihistamines; monotherapy/combined). We found that a single probiotic strain (LP-33) can significantly improve the quality of life of patients with AR from the meta-analysis of three studies. Two studies used mixed probiotic strains. One study demonstrated that a Bifidobacteria mixture (*B. longum* BB536, *B. infantis* M-63, and *B. breve* M-16 V) was able to significantly improve AR symptoms and quality of life in children with pollen-induced AR and intermittent asthma (39). Another study showed that probiotic NVP-1703 (a mixture of *B. longum* and *L. plantarum*) relieves AR symptoms by prompting Treg cells to release IL-10 (42). However, there was a high heterogeneity from meta-analysis of two studies, which may be related to the use of different probiotics. The different strains of probiotics, doses, and durations may be the sources of heterogeneity. To date, no serious adverse events have been observed for probiotic treatment; thus, it appears to be safe.

To sum up, probiotic supplement seems to be effective in ameliorating AR symptoms and improving the quality of life, but there is high heterogeneity in some results after subgroup analysis, and clinicians should be cautious when recommending probiotics in treating AR.

There are some limitations in this meta-analysis. First, the sample size of some included RCTs was small. Second, airborne pollen concentrations are associated with symptom severity and recovery in patients with SAR. The pollen concentrations varied due to different regions in different trials. This is a source of clinical heterogeneity.

## Conclusion

This study found that in spite of the positive results of some outcomes, there is weak evidence that probiotics have a potential benefit in the treatment of AR. More RCTs using specific probiotic strains and consistent outcome measures are also needed in the future to investigate efficacy and safety.

## Data Availability Statement

The original contributions presented in the study are included in the article/**Supplementary Material**. Further inquiries can be directed to the corresponding author.

## Author Contributions

CL, X-DA, and ML were involved in the methodological design of the systematic review, and conducted the acquisition of data, analyses, and interpretation. SP directed and organized the systematic review and the methodologist team, was involved in the initial concept and methodological design of the systematic review, and conducted data acquisition and interpretation. ZL was involved in the initial concept and methodological design of the systematic review, conducted data interpretation, and provided substantial feedback on the drafted manuscript. CL wrote the manuscript and SP revised the manuscript. All authors contributed to the article and approved the submitted version.

## Funding

This project was funded by Foundation of Chengdu Science and Technology Bureau (No. 2021-YF05-01940-SN). The sponsors are not involved in the design, execution, or writing of the study.

## Conflict of Interest

The authors declare that the research was conducted in the absence of any commercial or financial relationships that could be construed as a potential conflict of interest.

## Publisher’s Note

All claims expressed in this article are solely those of the authors and do not necessarily represent those of their affiliated organizations, or those of the publisher, the editors and the reviewers. Any product that may be evaluated in this article, or claim that may be made by its manufacturer, is not guaranteed or endorsed by the publisher.

## Supplementary Material

Supplementary Material 1 Subgroup analysis according to classification of allergic rhinitis for allergic rhinitis symptoms score.

Supplementary Material 2 Subgroup analysis according to combination of drugs for allergic rhinitis symptoms score.

Supplementary Material 3 Subgroup analysis according to intervention of treatment group for allergic rhinitis symptoms score.

Supplementary Material 4 Subgroup analysis according to classification of allergic rhinitis for rhinoconjunctivitis quality of life questionnaire score.

Supplementary Material 5 Subgroup analysis according to combination of drugs for rhinoconjunctivitis quality of life questionnaire score.

Supplementary Material 6 Subgroup analysis according to intervention of treatment group for rhinoconjunctivitis quality of life questionnaire score.

Supplementary Material 7 Subgroup analysis according to classification of allergic rhinitis for total IgE.

Supplementary Material 8 Subgroup analysis according to combination of drugs for total IgE.

Supplementary Material 9 Subgroup analysis according to classification of allergic rhinitis for sIgE.

Supplementary Material 10 Subgroup analysis according to combination of drugs for sIgE.

Supplementary Material 11 Subgroup analysis according to classification of allergic rhinitis for Th1/Th2 ratio.

Supplementary Material 12 Subgroup analysis according to combination of drugs for Th1/Th2 ratio.
