# Supplementary figures and images for: The Efficacy and Safety of Probiotics for Allergic Rhinitis: A Systematic Review and Meta-Analysis
Source: Front Immunol. 2022 May 19;13:848279. doi: 10.3389/fimmu.2022.848279 (PMC9161695; doi:10.3389/fimmu.2022.848279)

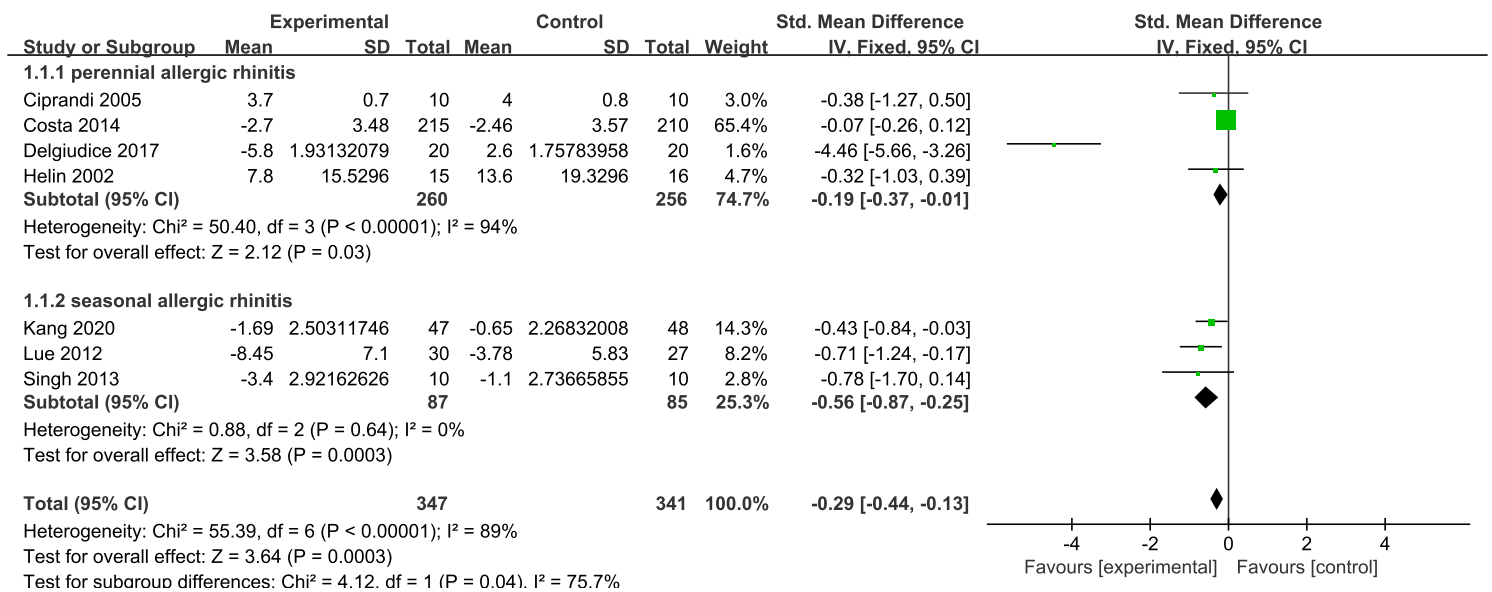

Supplement: Supplementary Material 1 — Subgroup analysis according to classification of allergic rhinitis for allergic rhinitis symptoms score. [file DataSheet_1.pdf]

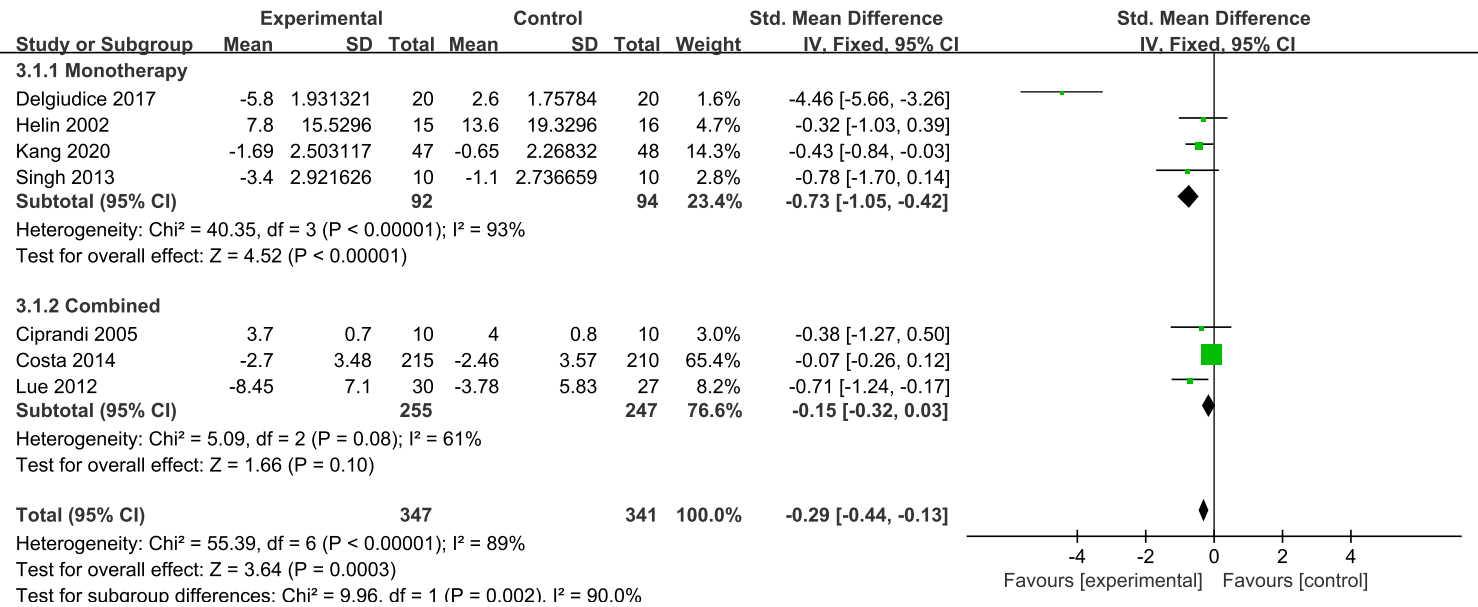

Supplement: Supplementary Material 2 — Subgroup analysis according to combination of drugs for allergic rhinitis symptoms score. [file DataSheet_2.pdf]

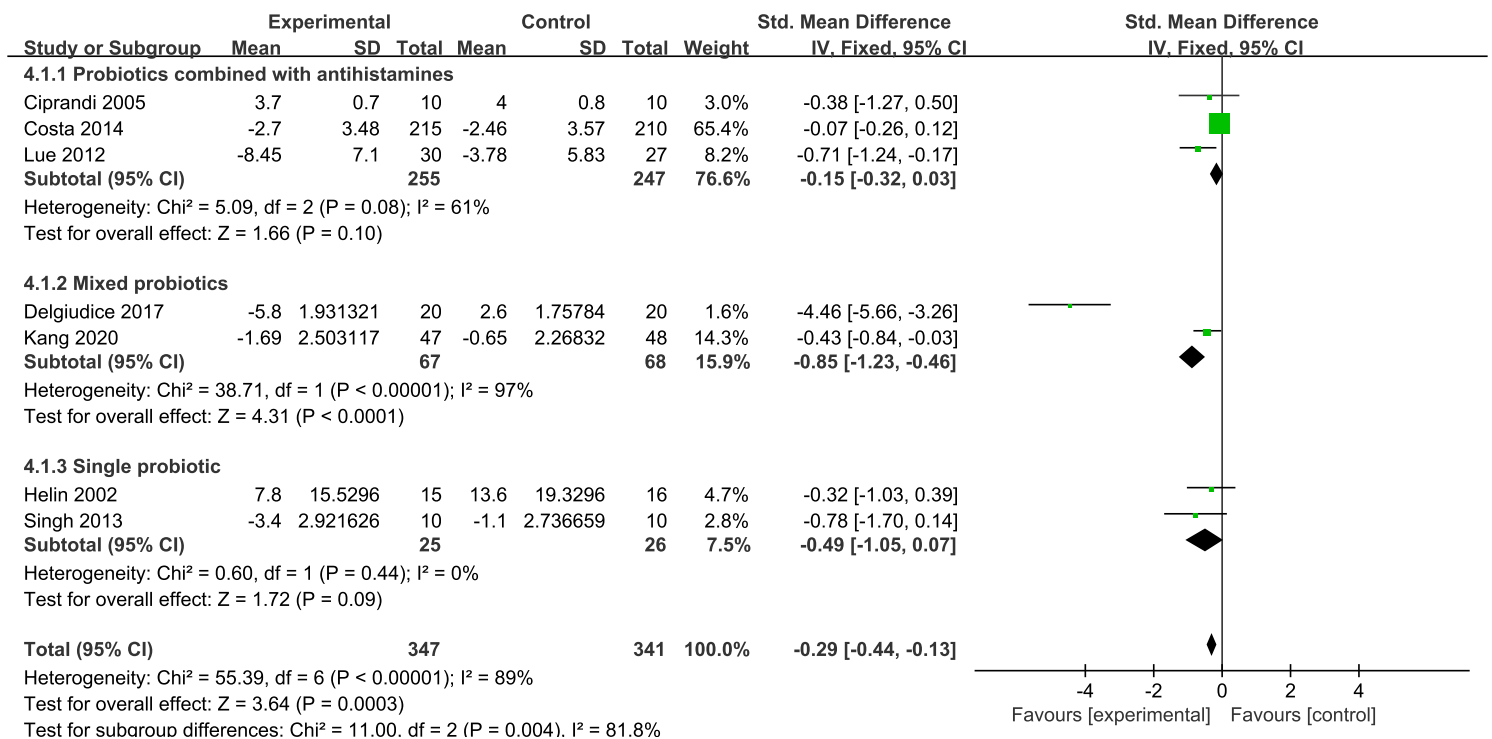

Supplement: Supplementary Material 3 — Subgroup analysis according to intervention of treatment group for allergic rhinitis symptoms score. [file DataSheet_3.pdf]

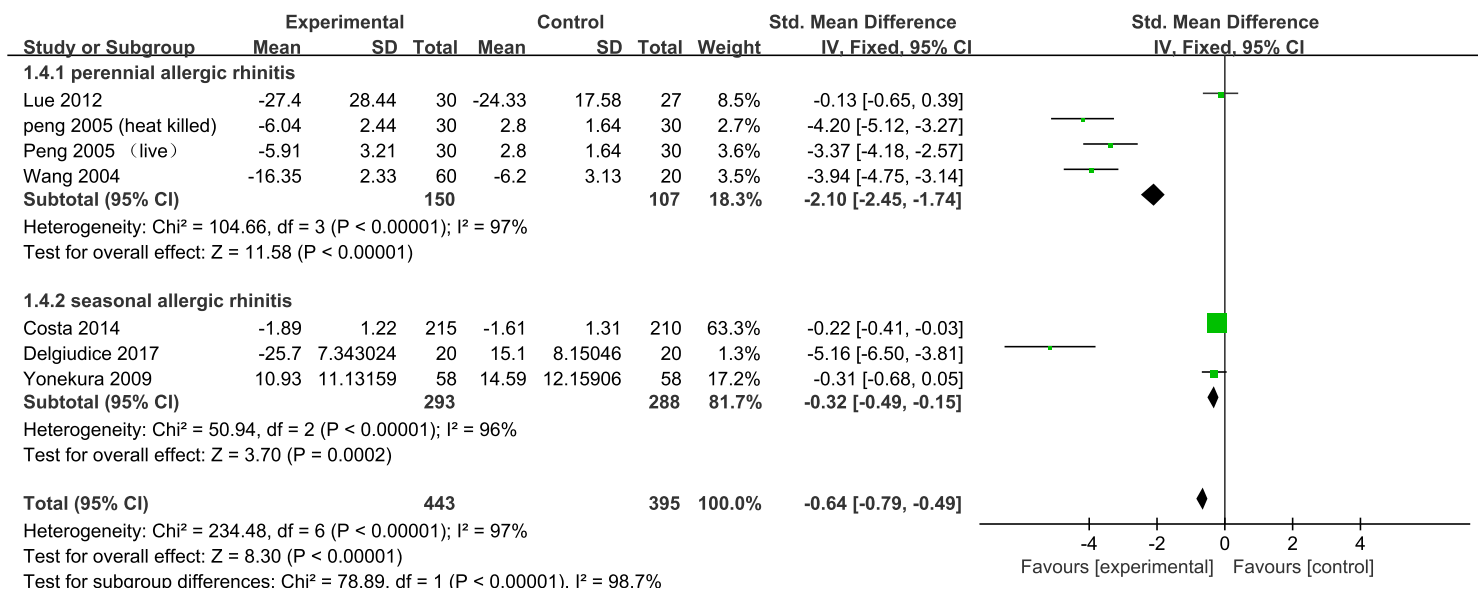

Supplement: Supplementary Material 4 — Subgroup analysis according to classification of allergic rhinitis for rhinoconjunctivitis quality of life questionnaire score. [file DataSheet_4.pdf]

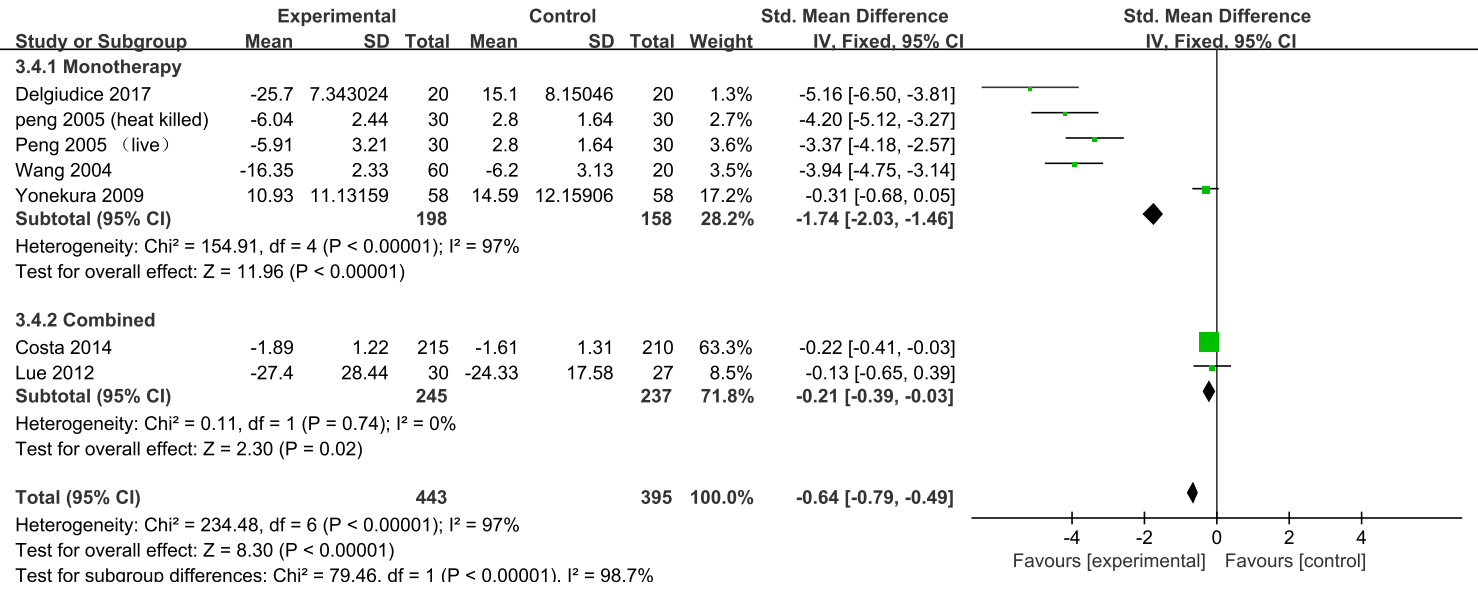

Supplement: Supplementary Material 5 — Subgroup analysis according to combination of drugs for rhinoconjunctivitis quality of life questionnaire score. [file DataSheet_5.pdf]

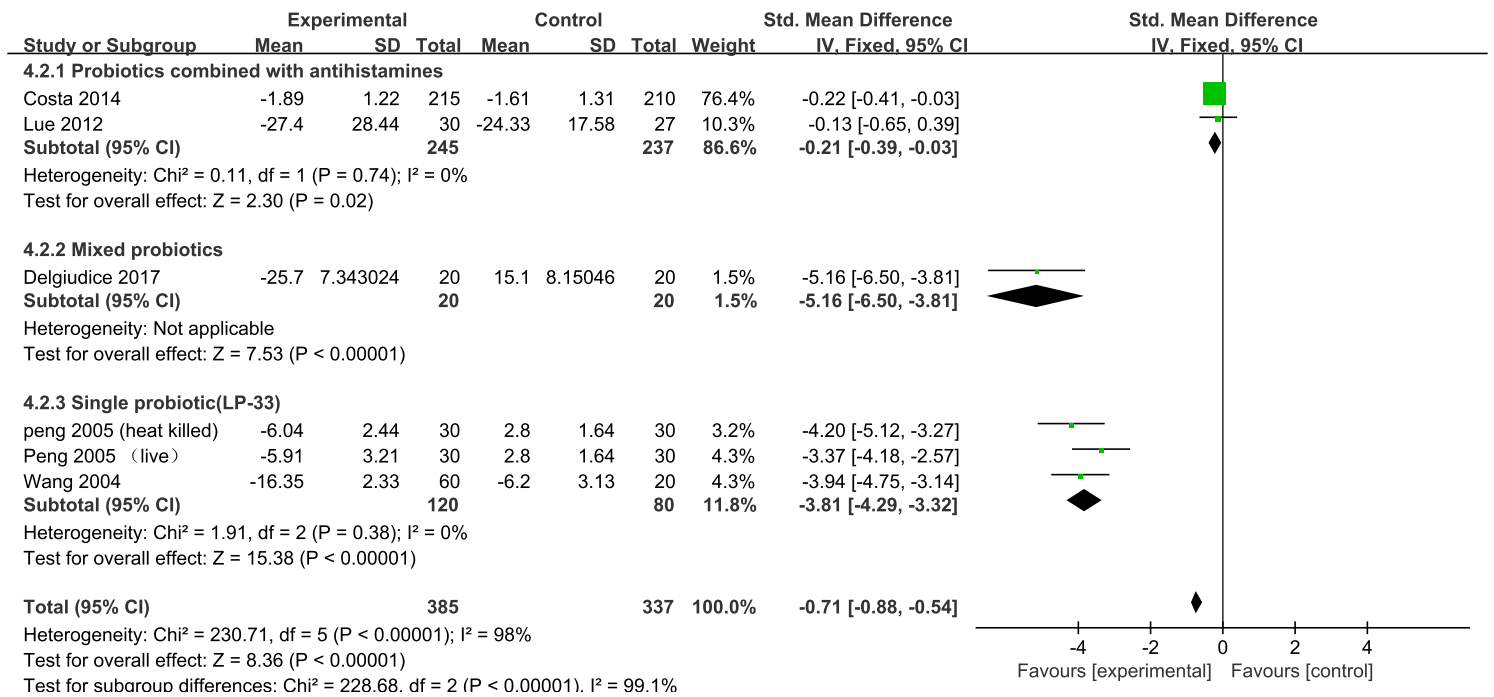

Supplement: Supplementary Material 6 — Subgroup analysis according to intervention of treatment group for rhinoconjunctivitis quality of life questionnaire score. [file DataSheet_6.pdf]

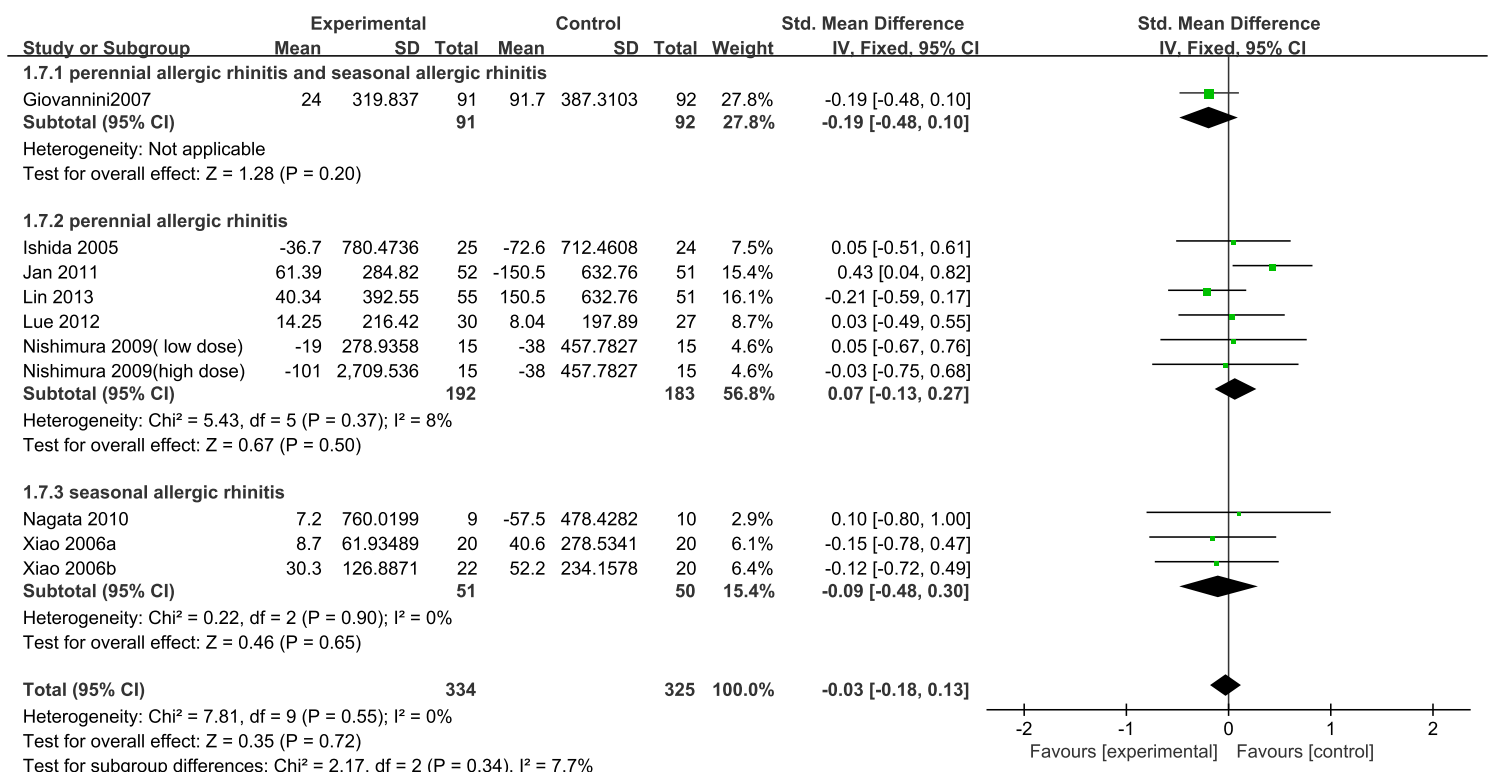

Supplement: Supplementary Material 7 — Subgroup analysis according to classification of allergic rhinitis for total IgE. [file DataSheet_7.pdf]

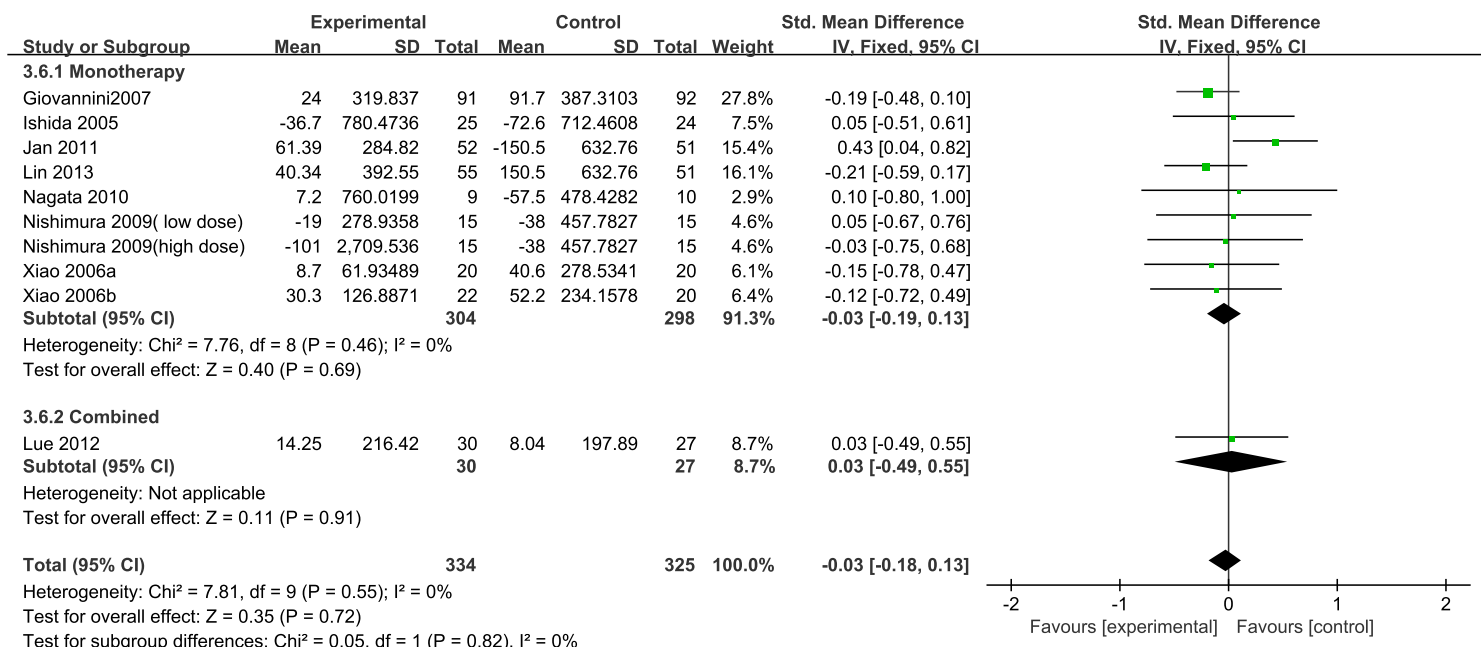

Supplement: Supplementary Material 8 — Subgroup analysis according to combination of drugs for total IgE. [file DataSheet_8.pdf]

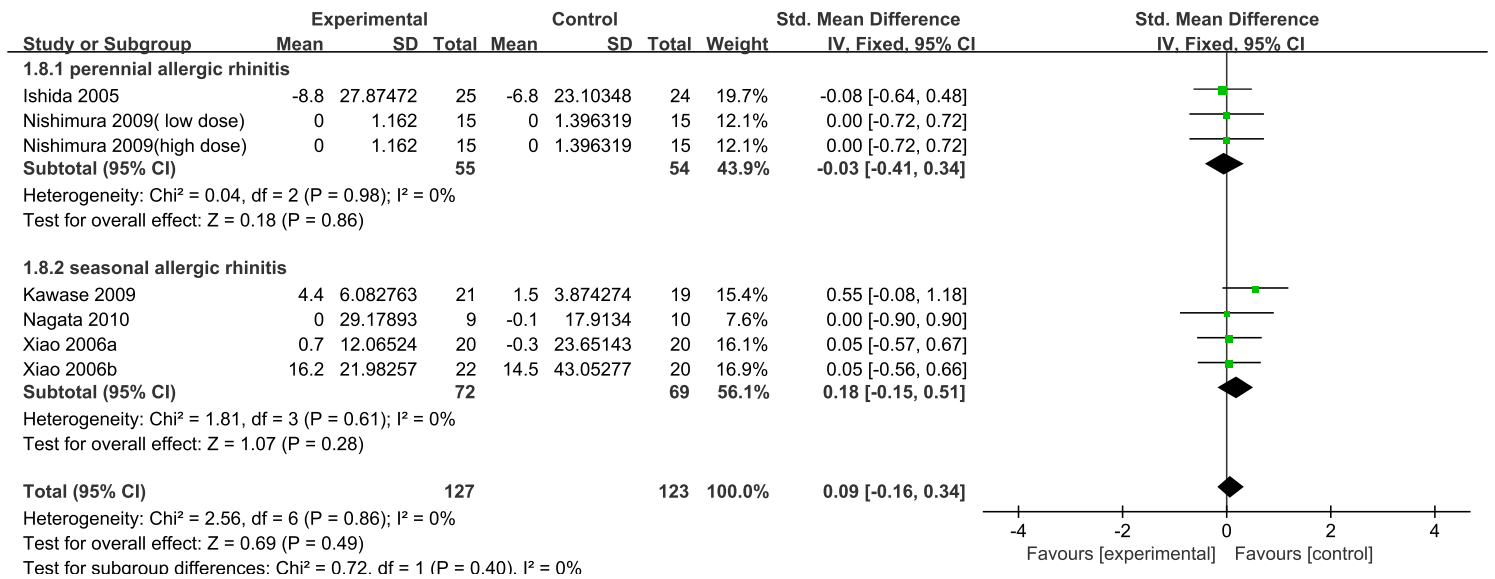

Supplement: Supplementary Material 9 — Subgroup analysis according to classification of allergic rhinitis for sIgE. [file DataSheet_9.pdf]

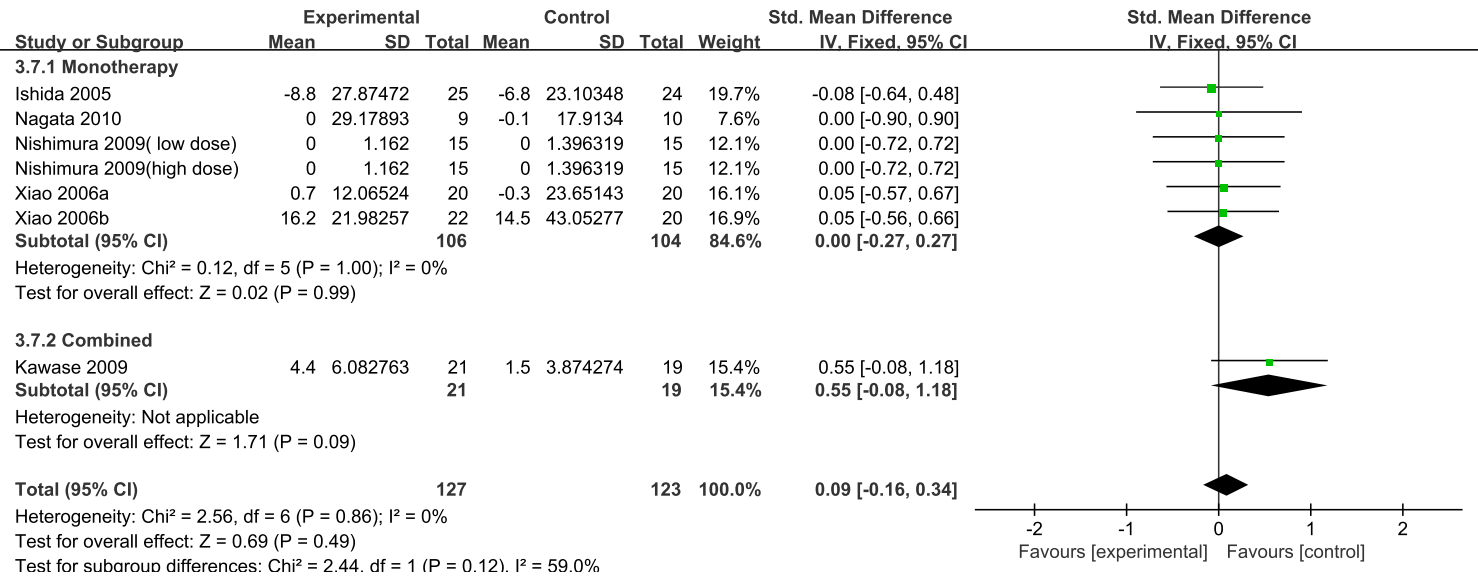

Supplement: Supplementary Material 10 — Subgroup analysis according to combination of drugs for sIgE. [file DataSheet_10.pdf]

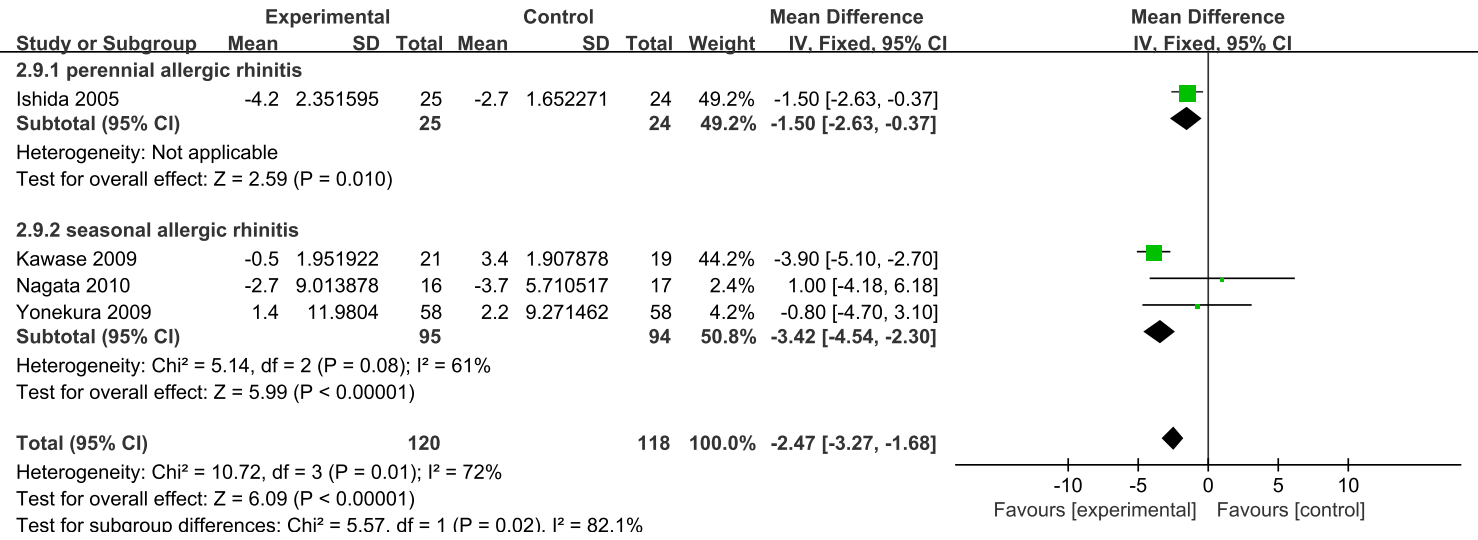

Supplement: Supplementary Material 11 — Subgroup analysis according to classification of allergic rhinitis for Th1/Th2 ratio. [file DataSheet_11.pdf]

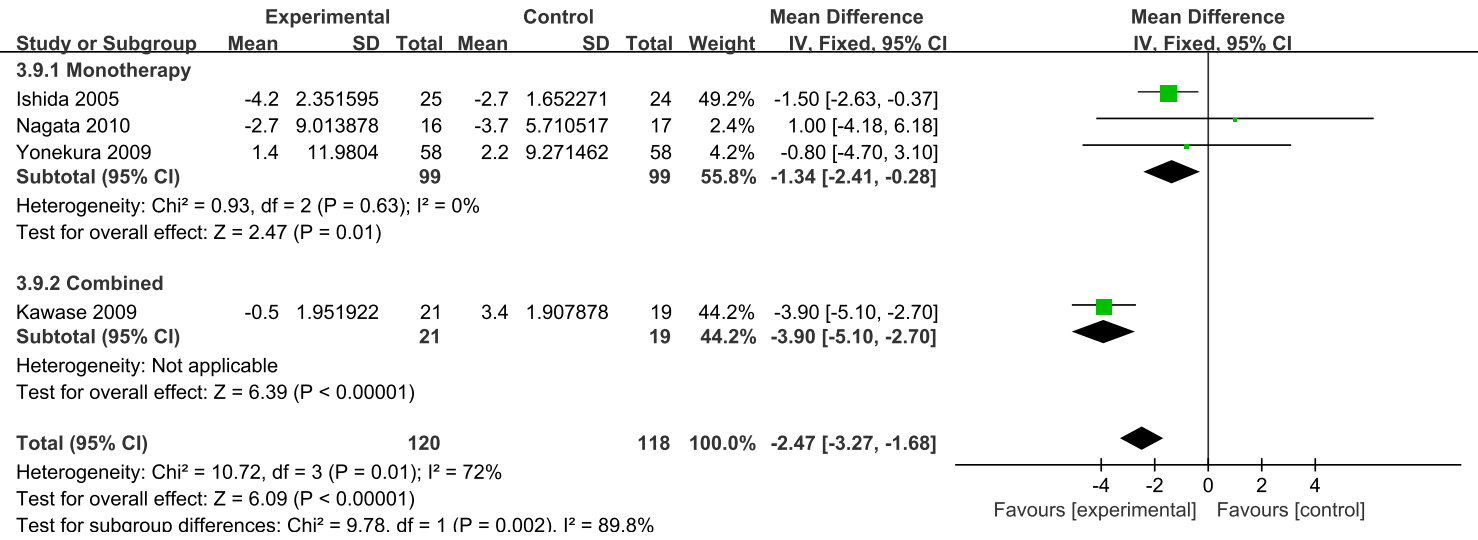

Supplement: Supplementary Material 12 — Subgroup analysis according to combination of drugs for Th1/Th2 ratio. [file DataSheet_12.pdf]
