# Supplementary material for: The Efficacy and Safety of Probiotics for Allergic Rhinitis: A Systematic Review and Meta-Analysis
Source: Front Immunol. 2022 May 19;13:848279. doi: 10.3389/fimmu.2022.848279 (PMC9161695; doi:10.3389/fimmu.2022.848279)
Supplement: Supplementary file 13 [file DataSheet_13.docx]

**Supplementary Material 13：Sensitivity analysis**

| **Remove one study** |  |  |  |
| --- | --- | --- | --- |
| **Allergic Rhinitis Symptoms Score** | **Std. Mean Difference (95%) (Fixed)** | ***P* value for overall effect** | ***I^2^*** |
| Ciprandi 2005 | -0.28 [-0.44, -0.13] | *P*=0.0004 | 91% |
| Costa 2014 | -0.70 [-0.96, -0.43] | *P*<0.0001 | 88% |
| Delgiudice 2017 | -0.22 [-0.37, -0.06] | *P*=0.006 | 39% |
| Helin 2002 | -0.28[-0.44, -0.13] | *P*=0.0004 | 91% |
| Kang 2020 | -0.26 [-0.43, -0.09] | *P*=0.002 | 91% |
| Lue 2012 | -0.25 [-0.41, -0.09] | *P*=0.002 | 91% |
| Singh 2013 | -0.27 [-0.43, -0.12] | *P*=0.0007 | 91% |
| **Rhino-conjunctivitis Quality of Life Questionnaire Score** | **Std. Mean Difference (95%) (Fixed)** | ***P* value for overall effect** | ***I^2^*** |
| Costa 2014 | -1.37 [-1.62, -1.12] | *P* < 0.00001 | 97% |
| Delgiudice 2017 | -0.58 [-0.74, -0.43] | *P* < 0.00001 | 97% |
| Lue 2012 | -0.69 [-0.85, -0.53] | *P* < 0.00001 | 98% |
| peng 2005 (heat killed) | -0.54 [-0.70, -0.39] | *P* < 0.00001 | 98% |
| Peng 2005 （live） | -0.54 [-0.70, -0.39] | *P* < 0.00001 | 98% |
| Wang 2004 | -0.52 [-0.68, -0.37] | *P* < 0.00001 | 97% |
| Yonekura 2009 | -0.71 [-0.88, -0.54] | *P* < 0.00001 | 98% |
| **Total IgE** | **Std. Mean Difference (95%) (Fixed)** | ***P* value for overall effect** | ***I^2^*** |
| Giovannini2007 | 0.04 [-0.15, 0.22] | *P*=0.7 | 0% |
| Ishida 2005 | -0.03 [-0.19, 0.13] | *P*=0.68 | 0% |
| Jan 2011 | -0.11 [-0.28, 0.06] | *P*=0.19 | 0% |
| Lin 2013 | 0.01 [-0.16, 0.17] | *P*=0.93 | 0% |
| Lue 2012 | -0.03 [-0.19, 0.13] | *P*=0.69 | 0% |
| Nagata 2010 | -0.03 [-0.19, 0.12] | *P*=0.69 | 0% |
| Nishimura 2009 (low dose) | -0.03 [-0.19, 0.13] | *P*=0.70 | 0% |
| Nishimura 2009(high dose) | -0.03 [-0.18, 0.13] | *P*=0.73 | 0% |
| Xiao 2006a | -0.02 [-0.18, 0.14] | *P*=0.81 | 0% |
| Xiao 2006b | -0.02 [-0.18, 0.14] | *P=*0.79 | 0% |
| **sIgE** | **Std. Mean Difference (95%) (Fixed)** | ***P* value for overall effect** | ***I^2^*** |
| Ishida 2005 | 0.13 [-0.15, 0.41] | *P*=0.37 | 0% |
| Kawase 2009 | 0.00 [-0.27, 0.27] | *P*=0.99 | 0% |
| Nagata 2010 | 0.09 [-0.17, 0.35] | *P*=0.48 | 0% |
| Nishimura 2009(low dose) | 0.10 [-0.17, 0.36] | *P*=0.46 | 0% |
| Nishimura 2009(high dose) | 0.10 [-0.17, 0.36] | *P*=0.46 | 0% |
| Xiao 2006a | 0.09 [-0.18, 0.37] | *P*=0.5 | 0% |
| Xiao 2006b | 0.09 [-0.18, 0.37] | *P*=0.5 | 0% |
| **Th1/Th2 ratio** | **mean difference (95%) (Fixed)** | ***P* value for overall effect** | ***I^2^*** |
| Nagata 2010 | -2.56 [-3.36, -1.75] | P < 0.00001 | 78% |
| Yonekura 2009 | -2.55 [-3.36, -1.73] | P < 0.00001 | 80% |
| Ishida 2005 | -3.42 [-4.54, -2.30] | P < 0.00001 | 61% |
| Kawase 2009 | -1.34 [-2.41, -0.28] | P = 0.01 | 0% |
